# Direct Observation of Interfacial Charge Transfer in Water Reduction at a Cu_2_O Photocathode by In Situ Near‐Field Heterodyne Transient Grating Spectroscopy

**DOI:** 10.1002/smsc.70384

**Published:** 2026-08-14

**Authors:** Seung Hyeon Jeong, Woon Yong Sohn

**Affiliations:** ^1^ Department of Chemistry Chungbuk National University Cheongju Korea; ^2^ Chungbuk National University G‐LAMP Project Group Cheongju Korea

**Keywords:** charge‐transfer dynamics, Cu_2_O photocathode, interfacial electron transfer, near‐field heterodyne transient grating, recombination

## Abstract

We investigated the interfacial charge‐carrier dynamics of a Cu_2_O photocathode during water reduction using near‐field heterodyne transient grating (NF‐HD‐TG) spectroscopy. Systematic measurements under various applied potentials, electrolyte conditions, and scavenger environments revealed multiple charge transfer and recombination processes, providing direct insight into reduction‐related electron dynamics. We were able to clearly observe signals arising from the interfacial electron transfer involved in the reduction process with a time constant of 1.33 × 10^−^^6^ s using NF‐HD‐TG spectroscopy at 0 *V*_RHE_, where the water reduction reaction occurs, indicating that the electron transfer governing the reduction kinetics is relatively faster than the hole transfer during water oxidation. To elucidate the origin of the relatively fast electron transfer, we obtained temperature‐dependent rate constants using NF‐HD‐TG spectroscopy, from which the activation energy was extracted. The results revealed that the rapid electron transfer originates from the relatively low activation energy of the electron‐transfer process.

## Graphical abstract

Near‐field heterodyne transient grating spectroscopy directly resolves reduction‐related electron dynamics at a Cu_2_O photocathode under water‐reduction conditions. A 1.33 μs interfacial electron‐transfer process is identified and validated by isotope‐effect measurements and complementary analyses, while temperature‐dependent measurements reveal a comparatively low activation barrier. The results uncover how surface‐trapped electrons drive water reduction.

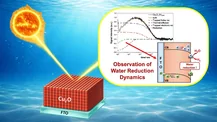

## 1 Introduction

Currently, global energy consumption still relies heavily on nonrenewable fossil fuels. Accordingly, the exploration and development of new renewable energy sources have emerged as an important and urgent challenge. Fujishima and Honda first demonstrated photoelectrochemical (PEC) water splitting, establishing the foundation for solar‐driven hydrogen production research [1]. Since then, PEC water splitting systems have emerged as a key technology for sustainable energy generation and have been continuously advanced through the development of efficient materials and systems [2, 3, 4, 5, 6, 7, 8, 9, 10, 11].

In PEC water‐splitting systems, photocathodes based on p‐type semiconductors play a crucial role in driving the water reduction reaction to produce hydrogen under illumination [12, 13, 14]. Various materials, including Cu_2_O, NiO, and GaP, have been extensively investigated as photocathodes [15, 16, 17]. In these systems, photocathodes operate in contact with the electrolyte, enabling solar‐driven hydrogen production through charge separation and interfacial charge‐transfer processes. Their performance is governed by the dynamics of photogenerated charge carriers, including carrier generation, bulk transport, surface accumulation, and interfacial charge‐transfer processes [18, 19, 20]. Accordingly, an understanding of charge‐carrier dynamics is essential for elucidating the intrinsic performance limitations of photoelectrodes and for guiding rational material and interface engineering.

To gain deeper insight into these processes, time‐resolved spectroscopic techniques provide powerful tools for elucidating charge‐carrier dynamics in PEC systems [21, 22, 23]. In particular, near‐field heterodyne transient grating (NF‐HD‐TG) spectroscopy, one of the time‐resolved spectroscopic techniques, employs heterodyne detection by mixing a strong reference field with a weak signal field, resulting in effective signal amplification and signal linearization enables exceptionally high detection sensitivity [24, 25]. These features allow the reliable detection of subtle refractive‐index changes induced by a very small population of charge carriers at the surface. In addition, the interfacial charges accumulate and generate a local electric field whose direction depends on the type of charge carrier, leading to opposite signal signs for electrons and holes [26]. This characteristic enables NF‐HD‐TG to clearly discriminate between electron‐ and hole‐related signals and thereby precisely identify the nature of accumulated charges at the photocathode–electrolyte interface.

In this work, we employ NF‐HD‐TG spectroscopy to systematically investigate the interfacial charge‐carrier dynamics of the Cu_2_O photocathode under water reduction conditions. By performing measurements under various electrochemical environments, including different applied potentials, electrolyte compositions, and scavenger conditions, we aim to gain a clearer understanding of the interfacial electron transfer and electron dynamics associated with the water reduction reaction. We identify interfacial electron transfer during the reduction process on a timescale of approximately 1.33 × 10^−^^6^ s, demonstrating that the electron transfer involved in the water reduction reaction is significantly faster than the hole transfer associated with water oxidation. Temperature‐dependent measurements further reveal that this fast interfacial electron transfer originates from its relatively low activation energy, providing mechanistic insight into interfacial electron transfer in a Cu_2_O photocathode.

## 2 Experimental

### 2.1 Preparation of Cu_2_O Photocathodes

Fluorine‐doped tin oxide (FTO) substrates (sheet resistance ~7 Ω sq^−1^, Sigma–Aldrich) were cleaned by sonication in a 1:1 (v/v) mixture of ethanol (Sigma–Aldrich) and acetone (Sigma–Aldrich) for 30 min to remove surface impurities. Cu_2_O films were subsequently deposited onto the cleaned FTO substrates via an electrodeposition process. The electrolyte solution was prepared using 0.3 M copper sulfate (CuSO_4_, Sigma–Aldrich) and 3 M lactic acid (C_3_H_6_O_3_, Sigma–Aldrich) as a chelating agent, and the pH of the solution was adjusted to 10.5 using sodium hydroxide (NaOH, Sigma–Aldrich). The mixed solution was heated and maintained at 60 °C, and Cu_2_O films were electrodeposited in a conventional three‐electrode configuration. The applied potential was −0.6 V versus Ag/AgCl for 500 s.

### 2.2 Characterization

Film morphologies were examined using scanning electron microscopy (SEM; Gemini 560, Carl Zeiss). The crystalline phases of the deposited films were identified by X‐ray diffraction (XRD) measurements performed with an X‐ray diffractometer (Miniflex 600, Rigaku). Optical absorption properties were investigated by recording UV–vis spectra over the wavelength range of 350–800 nm using a UV–vis spectrophotometer (Cary 8454, Agilent Technologies).

### 2.3 PEC Measurements

PEC measurements were carried out using a CHI650E electrochemical workstation under simulated solar irradiation (AM 1.5 G, 100 mW cm^−2^) provided by a xenon‐lamp solar simulator. Current density–potential (*J*–*V*) characteristics were obtained by linear sweep voltammetry (LSV). Photoelectrochemical impedance spectroscopy (PEIS) was also performed under 0 *V*_RHE_. A standard three‐electrode photoelectrochemical cell was employed, consisting of a Cu_2_O photocathode as the working electrode, a platinum counter electrode, and an Ag/AgCl reference electrode. An aqueous 0.1 M sodium sulfate solution (Na_2_SO_4_, Sigma–Aldrich) was used as the electrolyte. All measured potentials were converted to the reversible hydrogen electrode (RHE) scale according to the Nernst equation (Equation (1)), where the standard potential of the Ag/AgCl reference electrode (*E* ^0^_Ag/AgCl_) was taken as 0.197 V at 25 °C.

(1) $$E_{\mathrm{RHE}}=E_{\mathrm{Ag}/\mathrm{AgCl}}+0.059\mathrm{pH}+E_{\mathrm{Ag}/\mathrm{AgCl}}^{0}$$

### 2.4 Time‐Resolved Measurements

To elucidate the dynamics of photoinduced charge carriers in Cu_2_O photocathodes, NF‐HD‐TG spectroscopy was employed. The fundamental working principle of this technique has been reported in previous studies [24, 25]. In the present optical configuration, a transmission grating was placed in front of the sample to generate the reference field from the 0th‐order diffraction, while the transient grating formed on the sample produced the diffracted signal field. The third‐harmonic output of a Nd:YAG laser (355 nm, 0.9 mJ per pulse, 10 Hz) was used as the excitation source, while a continuous‐wave diode laser operating at 638 nm served as the probe beam. Illumination of the pump beam onto the thin‐film samples resulted in the formation of a transient grating, and both the diffracted signal and the reference beam corresponding to the 0th‐order diffraction were collected at the same detection position. The interference condition between the signal and reference beams was optimized by adjusting the distance between the sample and the transmission grating. Transient responses were detected using a photodiode (Thorlabs, PDA36A2). To suppress scattered pump light and fluorescence emitted from the excited samples, an edge filter (Semrock RazorEdge, LP02‐355RU, 355 nm) and a long‐pass filter (Thorlabs, FELH0600, 600 nm) were placed in front of the detector. The NF‐HD‐TG signals were monitored and recorded with an oscilloscope (Teledyne LeCroy, T3DSO3504). All measurements were conducted using the same three‐electrode configuration as that employed for the PEC experiments, under various electrolytes and applied potentials.

## 3 Results and Discussion

The crystal structure of Cu_2_O deposited on the FTO substrate was examined by X‐ray diffraction (XRD), and the results are shown in Figure S1. All diffraction peaks corresponding to Cu_2_O were clearly observed, confirming the successful formation of Cu_2_O on the FTO substrate [27, 28]. The surface SEM image of the Cu_2_O film is shown in Figure S2a, revealing a typical Cu_2_O morphology [28]. In addition, the cross‐sectional SEM image in Figure S2b indicates a film thickness of approximately 300 nm. The optical absorption properties of the Cu_2_O photocathode were evaluated by UV–vis spectroscopy in Figure S3a, showing strong absorption in the visible‐light region. The optical bandgap was estimated from the corresponding Tauc plot in Figure S3b, yielding a value of 2.06 eV, which is consistent with the reported bandgap of Cu_2_O [29]. LSV was performed to investigate the cathodic photocurrent behavior of the Cu_2_O photocathode. As shown in Figure S4a, a pronounced reduction current was observed under illumination, with a photocurrent density of approximately −0.20 mA cm^−2^ at 0 *V*_RHE_. In addition, a plateau is observed in the LSV curve of the H_2_O‐based electrolyte in the potential range of 0.1–0.2 *V*_RHE_. This behavior is commonly observed in aqueous electrolytes and can be attributed to a diffusion‐limited regime, where the current is governed by mass transport [30]. The semiconductor properties of the Cu_2_O film were further evaluated by Mott–Schottky (M–S) analysis. The M–S plot exhibited a negative slope, confirming the p‐type nature of Cu_2_O, as shown in Figure S4b.

We performed NF‐HD‐TG spectroscopy to investigate the photoexcited charge‐carrier dynamics of the Cu_2_O photocathode. Since the primary objective of this study is to elucidate the behavior of surface‐trapped electrons governing the water reduction reaction, accurate identification of electron‐related components is essential. The measured NF‐HD‐TG responses contain rising and decay components that reflect the temporal evolution of refractive index changes induced by charge accumulation at the interface because this method is highly sensitive to the photoelectrode–electrolyte interface [24, 25]. In general, electrons and holes generate local electric fields in opposite directions via the linear electro‐optic (Pockels) effect, resulting in refractive index changes of opposite sign [26]. Consequently, electron‐ and hole‐related signals are superimposed in the NF‐HD‐TG responses with opposite signs, contributing simultaneously to both the rising and decay processes. In addition, the NF‐HD‐TG technique used in this study measures transient changes in the refractive index that originate from local electric fields induced by charge carriers. In this context, charge carriers trapped at surface states generate strong, localized electric fields, which lead to significant refractive index modulation and thus dominate the observed signal. In contrast, free charge carriers are spatially delocalized and therefore produce much weaker local electric field gradients, resulting in a negligible contribution to the refractive index change. Consequently, the NF‐HD‐TG method is inherently more sensitive to trapped charge carriers than to delocalized free charge carriers. Based on this principle, we interpret the observed dynamics as predominantly reflecting surface‐trapped charge carriers. Therefore, the reduction process discussed in this work is most reasonably attributed to surface‐state electrons that are populated via transfer from the conduction band, rather than direct reactions of fully delocalized electrons.

Therefore, the NF‐HD‐TG responses were fitted to the sum of two positive and two negative exponential functions, as described in Equation (2) . In this fitting procedure, the positive exponential components correspond to decay signals, whereas the negative exponential components correspond to rising signals, allowing the individual temporal characteristics of the NF‐HD‐TG responses to be described. The number of exponential terms was chosen as the minimal set required to adequately reproduce both the rising and decay features of the experimental data, while avoiding overfitting.

(2) $$\begin{array}{cc} I\left(t\right) & =A_{1}\exp(-t/\tau_{1})+A_{2}\exp(-t/\tau_{2})\\ & \;-A_{3}\exp(-t/\tau_{3})-A_{4}\exp(-t/\tau_{4}) \end{array}$$

Prior to the component analysis, the extremely fast decay signals observed in the early‐time regime of certain responses were excluded from the fitting procedure. To identify the origins of each component, we first examined whether a thermal diffusion component varying with the grating spacing (*Λ*) was present. The NF‐HD‐TG responses of the Cu_2_O photocathode measured at different *Λ* are presented in Figure S5; the corresponding time constants are summarized in Table S1. The analysis revealed that the time constant of a specific component strongly depended on *Λ*, which is consistent with the characteristics of thermal diffusion. Accordingly, the first component, $A_{1}\exp(-t/\tau_{1})$, can be assigned to a decay process arising from thermal diffusion. At *Λ* = 30 μm, the timescale of thermal diffusion overlapped with that of the rising process, making reliable identification of the rising component difficult. Consequently, all subsequent measurements were performed at *Λ* = 80 μm, where the temporal overlap was sufficiently reduced to allow clear discrimination of the dynamics hidden in the rising part.

**FIGURE 1:**
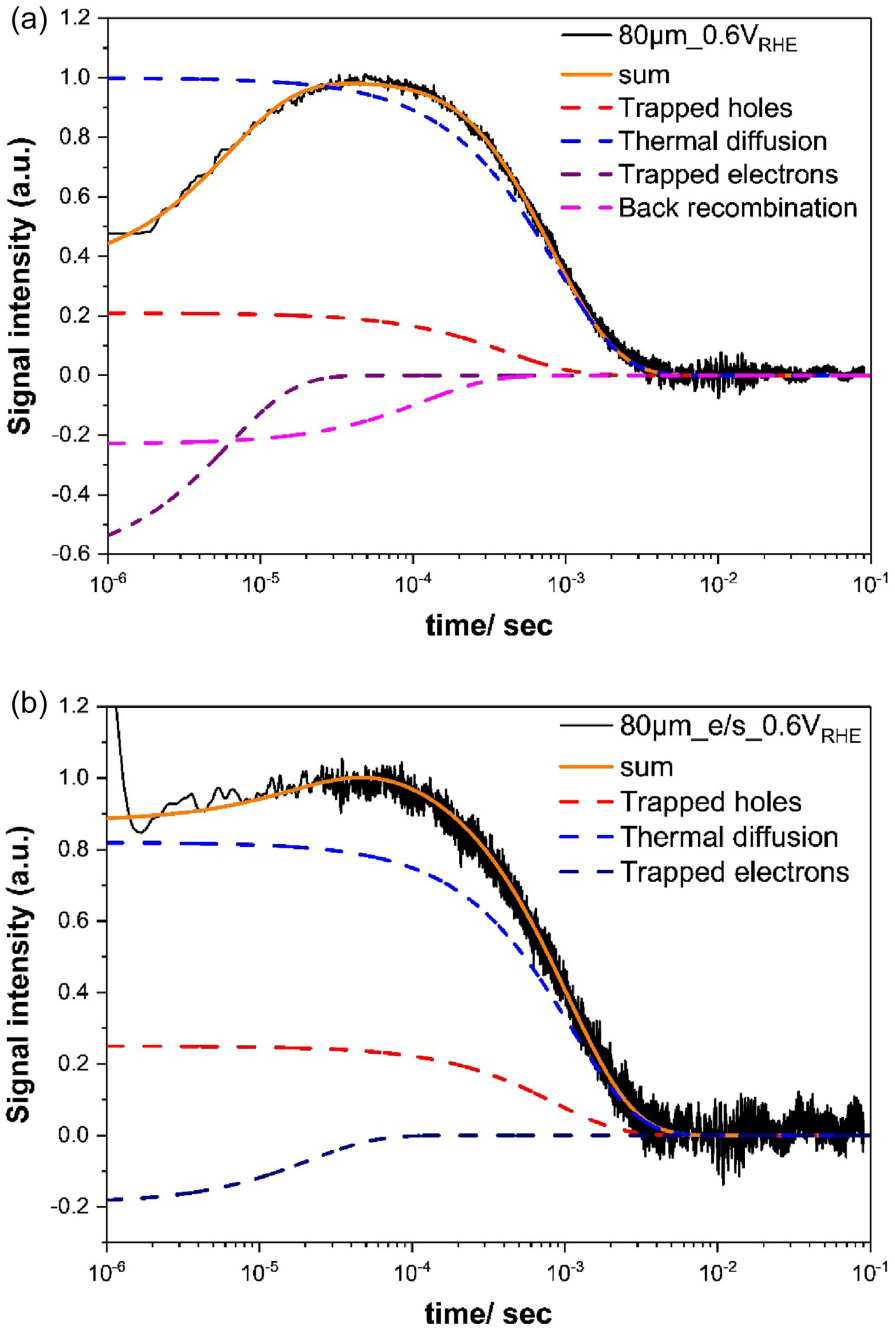
NF‐HD‐TG responses of the Cu_2_O photocathode at 0.6 *V*_RHE_ with a grating spacing of 80 μm, along with the corresponding fitting curves (a) in 0.1 M Na_2_SO_4_ electrolyte solution and (b) with 0.1 M Na_2_S_2_O_8_ added as an electron scavenger in the electrolyte.

The second component also appears as a decay feature in the NF‐HD‐TG responses. To determine whether this response originates from either hole‐ or electron‐related processes, measurements were conducted at 0.6 *V*_RHE_ under two electrolyte conditions: (a) 0.1 M Na_2_SO_4_, (b) 0.1 M Na_2_SO_4_ containing 0.1 M sodium persulfate (Na_2_S_2_O_8_) as an electron scavenger (e/s) [31], as shown in Figure 1. The extracted time constants and amplitudes are summarized in Tables 1 and 2, respectively. Upon addition of the e/s, the time constant ($\tau_{2}$) increased from 3.34 × 10^−^^4^ s to 8.51 × 10^−^^4^ s, and the corresponding amplitude $\left(A_{2}\right)$ slightly increased from 0.21 to 0.25. These changes indicate that the dynamics of this component became slower and the amount of the surface‐trapped charge carriers related to this process increased. The e/s captures interfacial electrons, thereby decreasing the population of free electrons in the conduction band. Therefore, the increase in the $\tau_{2}$ can be interpreted as a result of the suppression of recombination due to the reduced population of free electrons, which leads to an increase in the effective lifetime of the surface‐trapped holes. The increase in the $A_{2}$ can be understood as originating from the enhanced accumulation of the photo‐generated holes at the surface due to the suppression of the recombination occurring on a faster time scale. Taken together, these results indicate that the second component originates from a process associated with surface‐trapped holes.

**TABLE 1:** List of time constants of each component.

	$\tau_{1}$ (×10^−^^4^ s)	$\tau_{2}$ (×10^−^^4^ s)	$\tau_{3}$ (×10^−^^6^ s)	$\tau_{4}$ (×10^−^^6^ s)	
(a) 0.1 M Na_2_SO_4_	8.70	3.34	6.21		120
(b) With e/s	11.0	8.51	21.1		

**TABLE 2:** Fitted amplitudes of each component.

	$A_{1}$	$A_{2}$	$A_{3}$	$A_{4}$
(a) 0.1 M Na_2_SO_4_	0.99	0.21	0.65	0.22
(b) With e/s	0.82	0.25	0.19	

The third and fourth components appear as rising signals in the NF‐HD‐TG responses. In the absence of scavengers, two rising components were clearly extracted from the response, whereas under the e/s conditions, only one rising component was found with a reduced intensity. Because the e/s captures electrons accumulated at the interface, the electron‐induced refractive‐index change is suppressed. The disappearance of one rising component and the decrease in the amplitude in the other are therefore consistent with their assignment to electron‐related processes. However, under e/s conditions, it is not possible to unambiguously make a one‐to‐one correspondence between the remaining rising component and the original third or fourth component.

The rising components exhibit a sign opposite that of the second (decay) component assigned to surface‐trapped holes. As discussed above, owing to the Pockels effect, charge accumulations of electrons and holes induce refractive‐index changes with opposite signs. Therefore, the opposite sign of the rising components further supports their assignment to electron‐mediated processes and, in turn, provides additional confirmation of the origin of the second component, the surface‐trapped holes.

**FIGURE 2:**
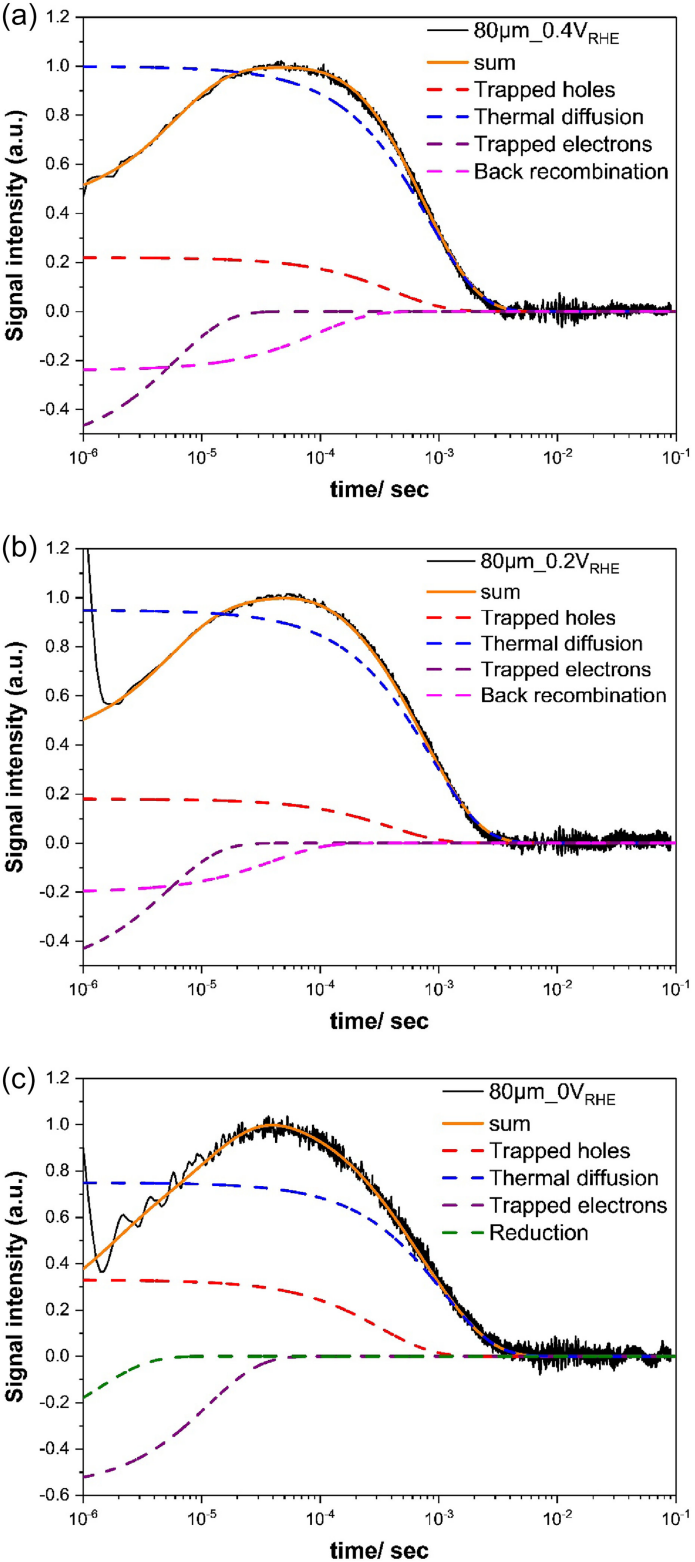
NF‐HD‐TG responses of the Cu_2_O photocathode measured at applied potentials of (a) 0.4, (b) 0.2, and (c) 0 *V*_RHE_.

Additional NF‐HD‐TG measurements were carried out at applied potentials of 0.4, 0.2, and 0 *V*_RHE_ to examine the potential‐dependent behavior of each component. The resulting responses are presented in Figure 2, while the corresponding time constants and amplitudes are summarized in Tables 3 and 4, respectively. Notably, a distinct change in the carrier dynamics was observed at 0 *V*_RHE_, where water reduction takes place under a strong built‐in electric field in the photocathode. The $\tau_{3}$ significantly increased, whereas that of the $\tau_{4}$ drastically decreased. At 0 *V*_RHE_, the photo‐generated holes drift toward the FTO substrate while the surface‐trapped electrons are rapidly consumed by the interfacial reduction reaction. As a result, the recombination between surface‐trapped electrons and free holes is suppressed, leading to an increase in the apparent time constant. This behavior is consistent with the delayed $\tau_{3}$, indicating that the third component corresponds to recombination between surface‐trapped electrons and free holes. Accordingly, the significantly decreased $\tau_{4}$ at 0 *V*_RHE_ is attributed to interfacial electron transfer associated with the reduction reaction, indicating that this process becomes dominant over back recombination under strongly cathodic conditions. As shown in Figure S4a, 0.6 *V*_RHE_ corresponds to a potential at which no photocurrent is generated, and only back recombination between holes transferred to the FTO substrate and photogenerated electrons at the Cu_2_O surface can occur. As the potential is shifted toward 0 *V*_RHE_, however, the surface‐trapped electrons are preferentially consumed via interfacial electron transfer for the reduction reaction rather than through back recombination. This preferential consumption of electrons leads to a marked acceleration of the kinetics related to $\tau_{4}$.

**TABLE 3:** List of time constants of each component.

	$\tau_{1}$ (×10^−^^4^ s)	$\tau_{2}$ (×10^−^^4^ s)	$\tau_{3}$ (×10^−^^6^ s)	$\tau_{4}$ (×10^−^^6^ s)	
(a) 0.4 *V*_RHE_	8.34	4.21	5.95		95.0
(b) 0.2 *V*_RHE_	8.70	3.89	5.24		39.8
(c) 0 *V*_RHE_	11.0	3.25	11.4		1.33

**TABLE 4:** Fitted amplitudes of each component.

	$A_{1}$	$A_{2}$	$A_{3}$	$A_{4}$
(a) 0.4 *V*_RHE_	0.99	0.21	0.54	0.24
(b) 0.2 *V*_RHE_	0.95	0.18	0.55	0.20
(c) 0 *V*_RHE_	0.75	0.33	0.57	0.38

The decrease in $\tau_{2}$ observed in Table 3 can be attributed to the enhanced band bending under the applied bias, which leads to the accumulation of electrons in the conduction band near the interface. The increased population of conduction band electrons raises the probability of recombination with trapped holes at the surface, thereby accelerating the recombination kinetics. As a result, *τ*_2_ decreases. This trend is consistent with the more pronounced accumulation of conduction band electrons at increasingly cathodic potentials, which further promotes recombination originating from the surface‐trapped holes.

We conducted additional experiments using D_2_O as the solvent to induce an isotope effect on the reduction reaction to verify that the accelerated $\tau_{4}$ observed at 0 *V*_RHE_ originates from the interfacial electron transfer for reduction processes. LSV measurements in the D_2_O‐based electrolyte showed a decrease in photocurrent density and a negative shift in the onset potential, as shown in Figure S6. This behavior is attributed to slower reduction kinetics resulting from the higher dissociation energy of O─D bonds [32].

**FIGURE 3:**
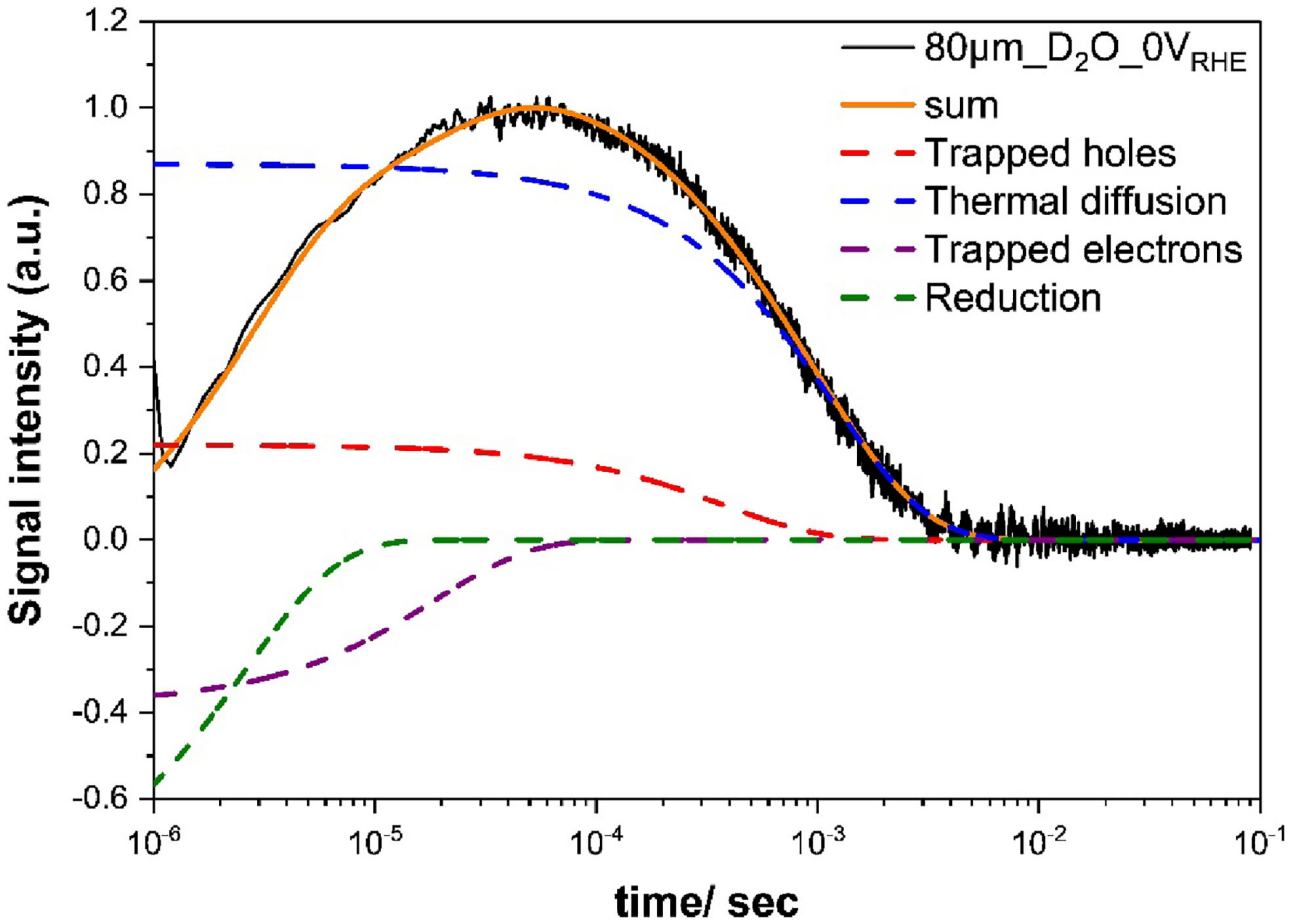
NF‐HD‐TG responses of the Cu_2_O photocathode measured at 0 *V*_RHE_ in a D_2_O‐based electrolyte containing 0.1 M Na_2_SO_4_.

Based on these results, NF‐HD‐TG measurements were carried out using a D_2_O‐based electrolyte containing 0.1 M Na_2_SO_4_. The resulting NF‐HD‐TG responses are presented in Figure 3, and the corresponding time constants of each component are summarized in Table 5. Consistent with the isotope effect, the $\tau_{4}$ at 0 *V*_RHE_ exhibits clear retardation when comparing H_2_O‐ and D_2_O‐based electrolytes. As summarized in Table 3c, the $\tau_{4}$ value obtained in the H_2_O‐based electrolyte is 1.33 × 10^−^^6^ s, whereas it is retarded to 2.54 × 10^−^^6 ^s in the D_2_O‐based electrolyte, as shown in Table 5. This retardation indicates slower interfacial electron‐transfer kinetics associated with water reduction reaction in D_2_O, further supporting that the $\tau_{4}$ component at 0 *V*_RHE_ originates from the electron transfer for the reduction process. In addition, the kinetic isotope effect derived from the measured $\tau_{4}$ values in the two electrolytes (*k*_H_/*k*_D_ ≈1.9) indicates that proton motion is directly involved in the electron‐transfer step. The magnitude of the isotope effect is consistent with proton‐coupled electron‐transfer theory (PCET) processes, where electron transfer is coupled to proton in the electrolyte. Such isotope effects arise from differences in zero‐point energy between O─H and O─D bonds, leading to slower proton‐associated dynamics in D_2_O [33]. Therefore, these results support that the reduction reaction is related to the PCET mechanism.

**TABLE 5:** List of time constants of each component.

	$\tau_{1}$ (×10^−^^4^ s)	$\tau_{2}$ (×10^−^^4^ s)	$\tau_{3}$ (×10^−^^6^ s)	$\tau_{4}$ (×10^−^^6^ s)	
0 *V*_RHE_ (D_2_O)	11.7	3.74	18.7		2.54

As summarized in Table 3c, the $\tau_{3}$ value in the H_2_O‐based electrolyte is 11.4 × 10^−6^ s, whereas it increases to 18.7 × 10−6 s in the D_2_O‐based electrolyte, as shown in Table 5. This increase can be attributed to the reduced population of free holes resulting from less efficient charge separation due to the isotope effect. The recombination process associated with $\tau_{3}$ corresponds to the recombination between surface‐trapped electrons and free holes and follows pseudo‐first‐order kinetics governed by the availability of free holes because the free hole concentration is significantly higher than that of surface trapped electrons. To elucidate the origin of the reduced free‐hole density, *M*–*S* analyses were performed in both H_2_O‐ and D_2_O‐based electrolytes for comparison. The results obtained in the H_2_O‐based electrolyte are shown in Figure S4b in Supporting Information, while those for the D_2_O‐based electrolyte are presented in Figure S7. In the H_2_O‐based electrolyte, the flat‐band potential (*V*_FB_) was determined to be 0.66 *V*_RHE_, whereas it decreased to 0.59 *V*_RHE_ in the D_2_O‐based electrolyte. This indicates a reduced band bending in the D_2_O system at 0 *V*_RHE_, which leads to a narrower depletion layer and a weaker internal electric field. Consequently, charge separation becomes less efficient, resulting in a lower population of free holes.

We performed PEIS analysis to figure out the origin of the inefficient charge separation of the Cu_2_O photocathode in the D_2_O‐based electrolyte. As shown in Figure S7, the Nyquist plots of the Cu_2_O photocathode and their corresponding fitting curves were obtained at 0 *V*_RHE_ in both H_2_O‐ and D_2_O‐based electrolytes. The impedance spectra were fitted using both an equivalent circuit with an ideal capacitance (*C*) and a constant phase element (CPE, *Q*) to account for non‐ideal interfacial behavior, and the corresponding fitting parameters (*R*_s_, *R*_1_, *R*_2_, *C*_1_, *C*_2_, *Q*_1_, and *Q*_2_) are summarized in Table S2, along with the circuit diagram presented in Figure S8. Here, *R*_s_ represents the total series resistance, *R*_1_ corresponds to the bulk resistance, and *R*_2_ denotes the interfacial resistance between the photoelectrode surface and the electrolyte. As shown in Table S2, the *R*_2_ values obtained from both C and Q fittings are consistently higher in the D_2_O‐based electrolyte than in the H_2_O‐based system, indicating less efficient interfacial charge transfer in D_2_O‐based electrolyte, which is consistent with the larger $\tau_{4}$ value in the D_2_O‐based electrolyte. Furthermore, in the D_2_O‐based electrolyte, the interfacial capacitance (*C*_2_) is lower than that in the H_2_O‐based electrolyte. This decrease indicates that fewer charges can accumulate at the electrode/electrolyte interface, reflecting a reduced ability to store photo‐generated carriers in the interfacial region. This indicates that replacing H_2_O with D_2_O affects the electrochemical double layer at the interface, which in turn appears to influence the surface properties of the photoelectrode. As a result, the strength of the local electric field within the depletion layer is diminished, making the separation of photogenerated electrons and holes less efficient, which is consistent with the reduced band bending at 0 *V*_RHE_ observed from the Mott–Schottky plots. Therefore, the free‐hole population in the Cu_2_O photocathode in D_2_O‐based electrolyte is reduced, which slows down the recombination rate and consequently leads to an increase in $\tau_{3}$.

**FIGURE 4:**
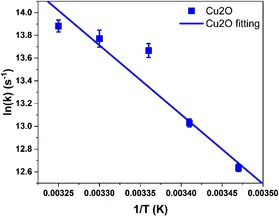
Arrhenius plots of Cu_2_O fitted using the Arrhenius equation. Error bars were obtained from two repeated measurements.

To determine the activation energy corresponding to the electron transfer for the water reduction, temperature‐dependent NF‐HD‐TG measurements were performed, and the corresponding Arrhenius analysis is presented in Figures S10 and S11, with the extracted values summarized in Tables S3 and S4. This analysis was conducted to elucidate why the electron transfer involved in the reduction reaction proceeds relatively faster than the hole transfer associated with water oxidation reported in the former study [34]. We determined the activation energy by constructing Arrhenius plots. The plotted data are shown in Figure 4, and the extracted parameters are summarized in Table 6. The obtained activation energy was 50.73 kJ mol^−1^, which is significantly lower than the charge‐transfer activation energy for the water oxidation reaction on the hematite photoanode reported in our previous study (103.8 kJ mol^−1^) [34], indicating that the energy barrier for the reduction reaction is relatively small. This low activation energy accounts for the remarkably fast charge‐transfer kinetics observed under reduction conditions.

**TABLE 6:** Activation energy and pre‐exponential factor value.

	Activation energy, kJ/mol	Pre‐exponential factor, s^−1^
Cu_2_O	50.73	5.03 × 10^14^

**FIGURE 5:**
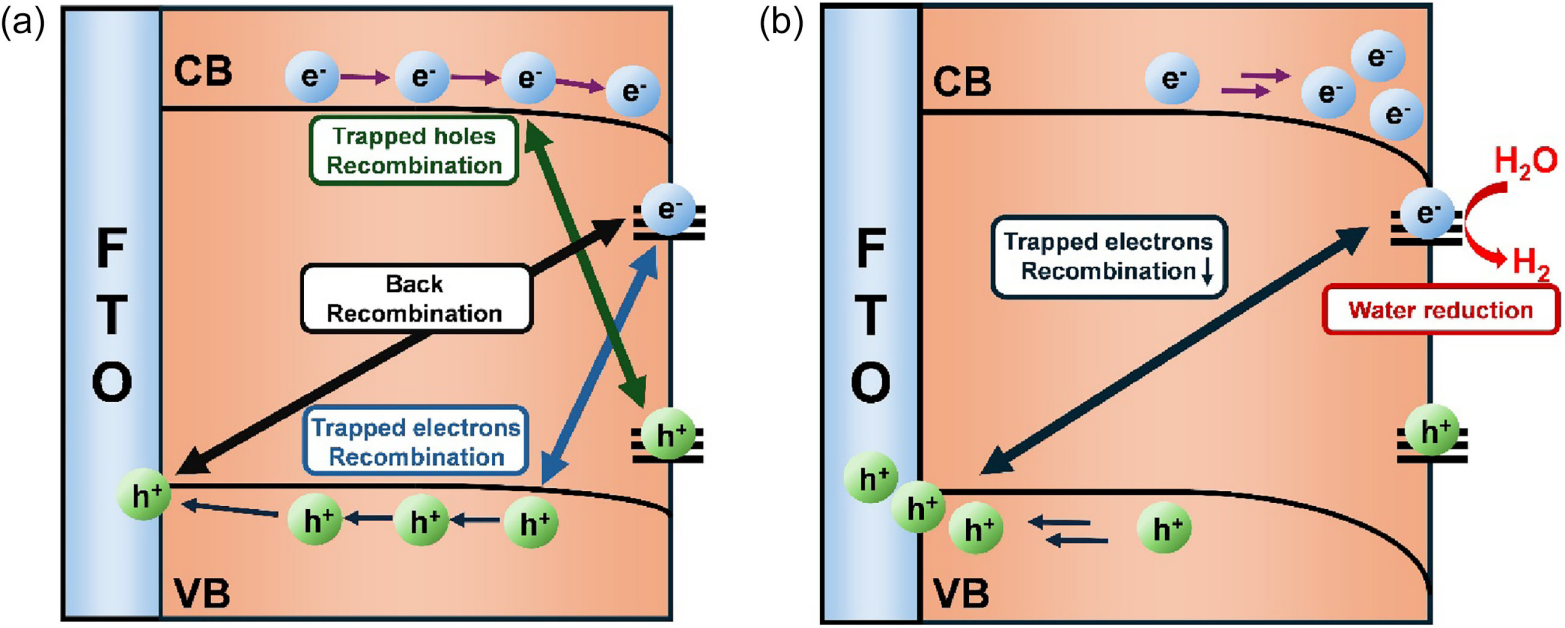
A schematic diagram of charge‐carrier dynamics in the Cu_2_O electrode, illustrating (a) the recombination process at 0.6 *V*_RHE_ and (b) the interfacial water reduction reaction process at 0 *V*_RHE_.

A summary of the charge‐carrier dynamics in the Cu_2_O photocathode is shown in Figure 5. Figure 5a illustrates the charge‐carrier dynamics at 0.6 *V*_RHE_, where no photocathodic current is generated. Upon light irradiation, photogenerated electrons and holes are produced, with electrons migrating toward the surface and holes toward the FTO conductive substrate. However, due to various factors such as defects, both electrons and holes are partially trapped at the surface. The recombination between trapped holes and conduction band electrons contributes to the decay component (second component). In addition, recombination between trapped electrons and valence band holes corresponds to the rising component (third component). Furthermore, surface‐trapped electrons can undergo back recombination with holes transferred to the FTO substrate, which is assigned to the fourth component. Figure 5b shows the charge‐carrier dynamics at 0 *V*_RHE_, where photocathodic current generation is possible. At this potential, enhanced band bending promotes more efficient charge separation. As a result, back recombination observed at longer time scales is inhibited, and the trap‐mediated electron recombination is retarded because photogenerated electrons are rapidly extracted to the interface. Meanwhile, an accelerated rising signal is detected, which is attributed to the interfacial electron transfer involved in the reduction reaction mediated by surface‐trapped electrons.

In this study, the opposite signs of the refractive index changes induced by photogenerated electrons and holes enabled us to clearly distinguish their respective contributions in the NF‐HD‐TG responses. Based on this characteristic, the electron‐ and hole‐related dynamics during the PEC reaction were independently analyzed, allowing us to directly determine the characteristic time constants during the reduction process. Considering that studies directly probing electron dynamics and their time‐resolved behavior in photocathodic reduction processes remain limited, the present approach provides valuable insight into both the mechanism and kinetics in a photocathode under reduction conditions. Therefore, this work demonstrates that NF‐HD‐TG spectroscopy is a powerful tool for elucidating charge‐carrier dynamics and reaction kinetics in photocathode systems, providing a useful platform for optimizing reduction reactions.

## 4 Conclusion

In this work, we systematically investigated the interfacial charge‐carrier dynamics of the Cu_2_O photocathode during water reduction using NF‐HD‐TG spectroscopy. By exploiting the opposite refractive index responses induced by photogenerated electrons and holes, their individual contributions to the transient signals were clearly separated, enabling direct analysis of reduction‐related electron processes. Through systematic control of applied potential, electrolyte composition, and scavenger conditions, multiple processes were clearly resolved. In particular, the reduction‐related time constant was precisely identified under water reduction conditions and further validated by isotope experiments using D_2_O. Temperature‐dependent measurements revealed a low activation energy, providing a quantitative explanation for the rapid electron‐transfer kinetics on the Cu_2_O photocathode. More broadly, this study demonstrates that NF‐HD‐TG spectroscopy offers a powerful and versatile platform for directly probing electron carrier dynamics in photocathodic systems. The methodology utilized here enables quantitative investigation of electron transfer kinetics and can be readily extended to other photocathode materials. This approach provides new opportunities for rational design and optimization of PEC devices for solar fuel production.

## Funding

This study was supported by Ministry of Education (RS‐2024‐00445180), Ministry of Science and ICT, South Korea (RS‐2025‐16066242).

## Conflicts of Interest

The authors declare no conflicts of interest.

## Supporting information


*J*–*V* curve, UV–vis spectra, *M* *–* *S* plot, PEIS, NF‐HD‐TG responses of the Cu_2_O photocathode with various grating spacings, applied potentials, and electrolyte conditions and the list of the time constants.

## Data Availability

The data that support the findings of this study are available from the corresponding author upon reasonable request.
