# Supplementary material for: Direct Observation of Interfacial Charge Transfer in Water Reduction at a Cu2O Photocathode by In Situ Near‐Field Heterodyne Transient Grating Spectroscopy
Source: Small Sci. 2026 Aug 14;6(8):e70384. doi: 10.1002/smsc.70384 (PMC13489322; doi:10.1002/smsc.70384)
Supplement: Supplementary file 1 [file SMSC-6-e70384-s001.pdf]

Supporting Information (SI)

Direct Observation of Interfacial Charge Transfer in Water Reduction at a Cu_2O Photocathode by In Situ Near-Field Heterodyne Transient Grating Spectroscopy

Seung Hyeon Jeong[1,2] and Woon Yong Sohn[1,2]*

1. Department of Chemistry, Chungbuk National University, Chungdae-ro 1, Cheongju, Chungbuk 28644, Korea

2. Chungbuk National University G-LAMP Project Group, Chungdae-ro 1, Cheongju, Chungbuk 28644, Korea

*Corresponding Author

W. Y. Sohn, Phone: 82-43-261-2285, E-mail: nunyong@chungbuk.ac.kr

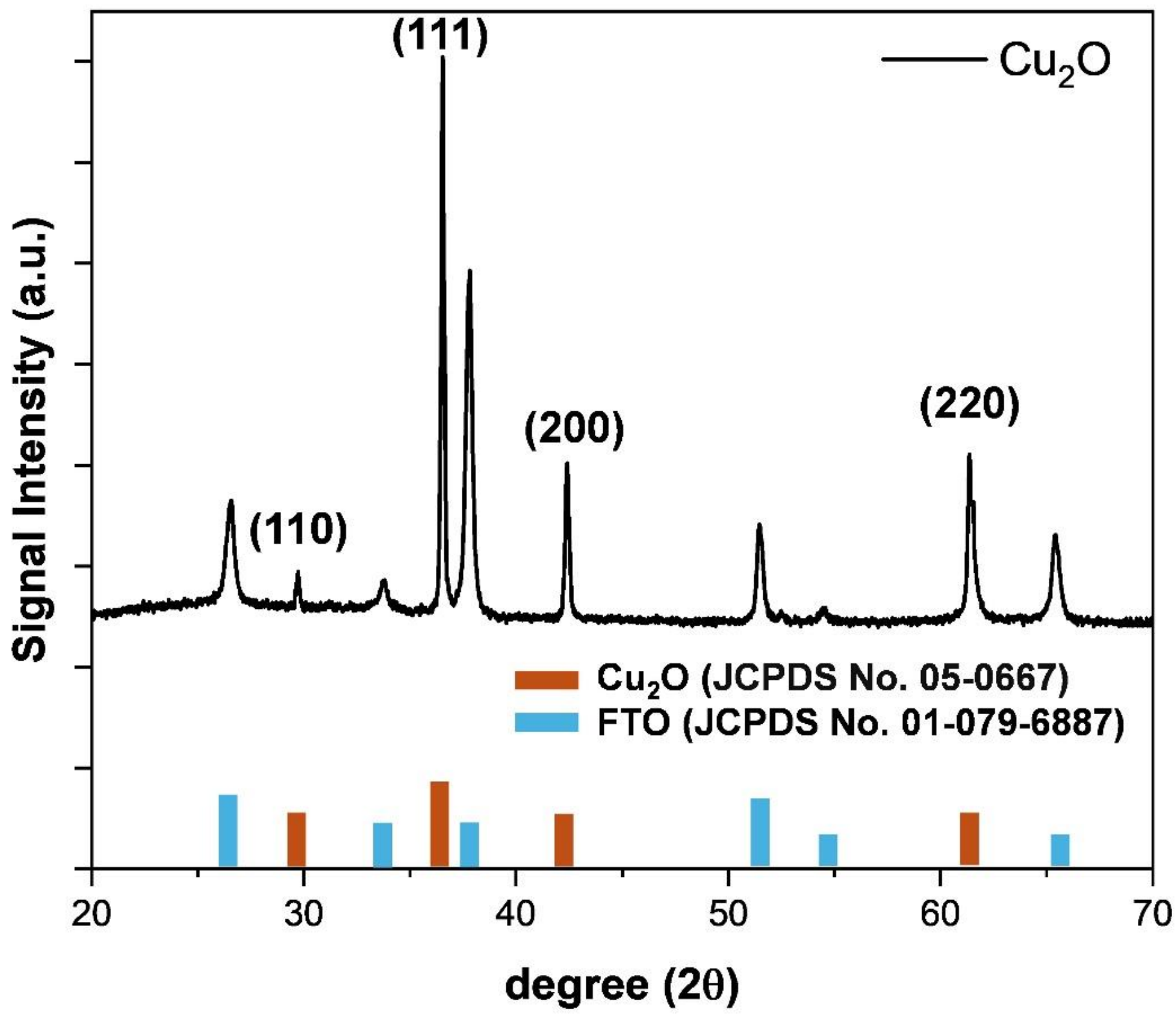

Figure S1 X-ray diffraction (XRD) pattern of Cu_2O deposited on an FTO substrate. The brown and blue bars indicate the reference diffraction patterns of Cu_2O (JCPDS No. 05-0667) and FTO (SnO_2, JCPDS No. 01-079-6887), respectively.

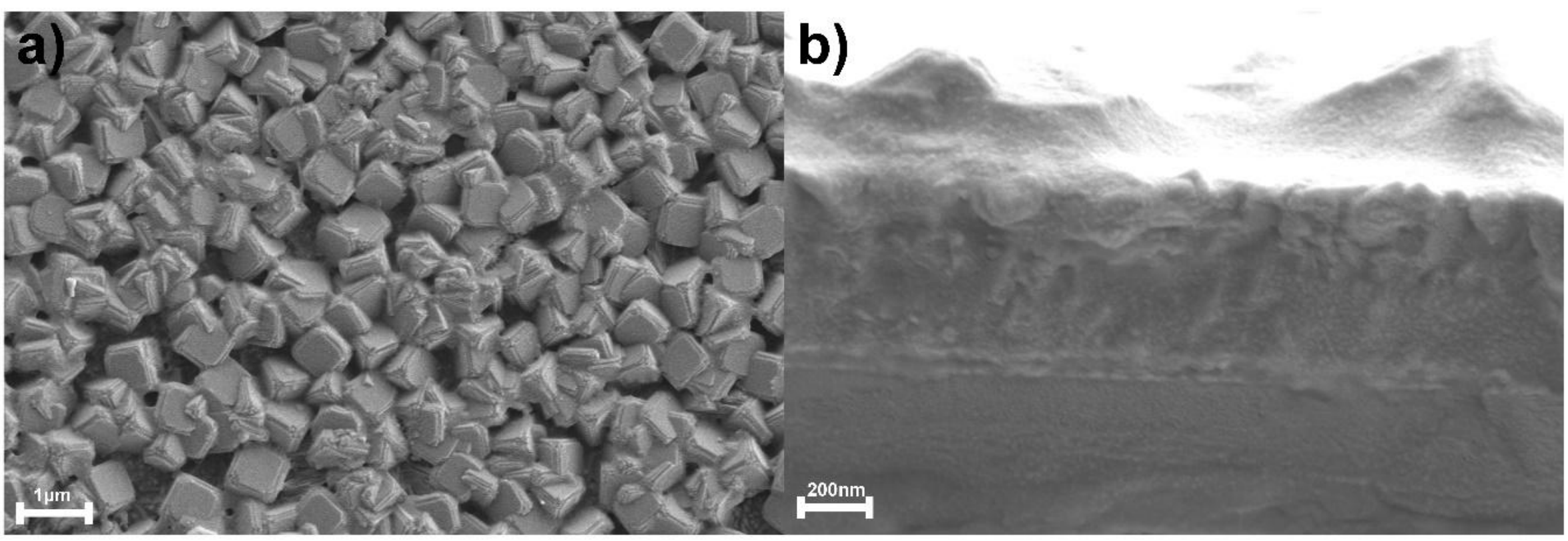

Figure S2. a) The top view and b) the cross view of SEM images of Cu_2O photocathode.

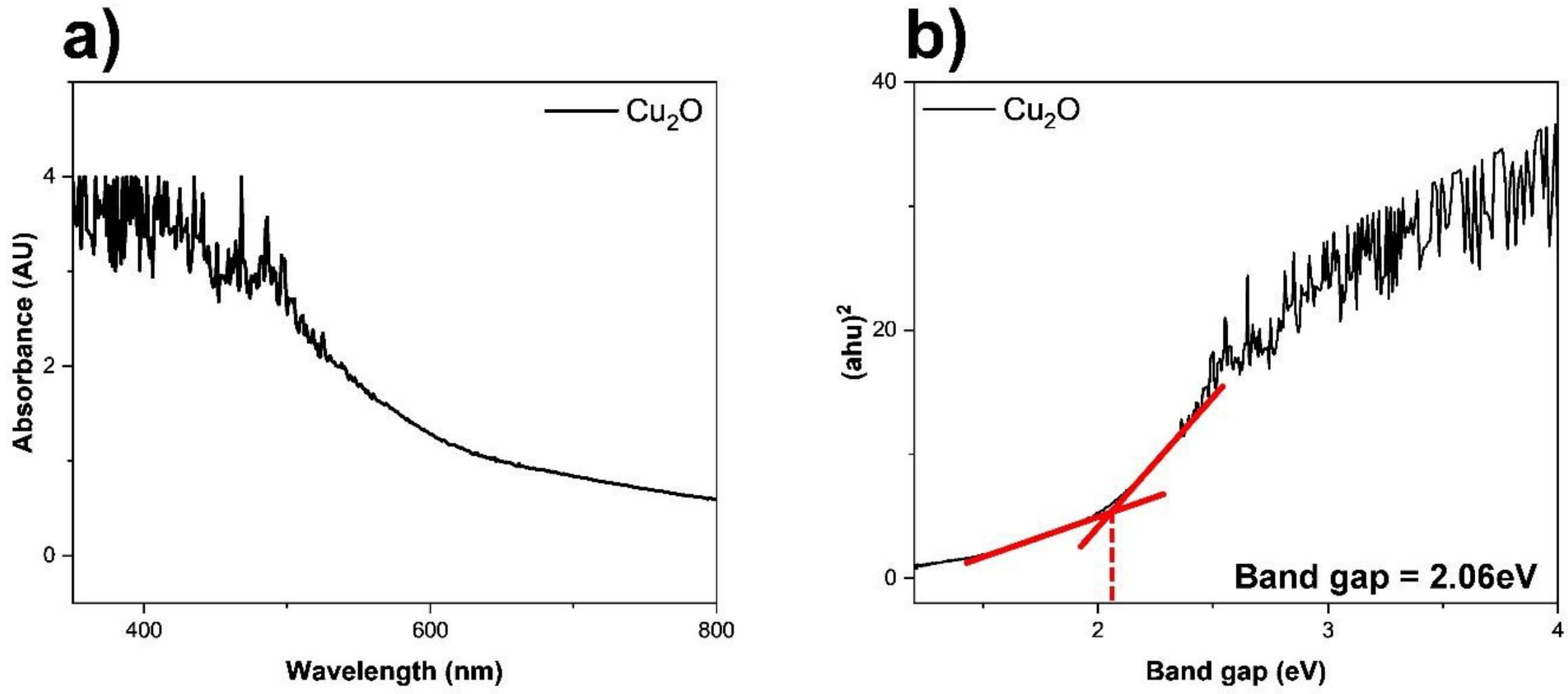

Figure S3. UV-vis spectra, ranging from 350 nm to 800 nm, and b) Tauc plots of Cu_2O photocathode.

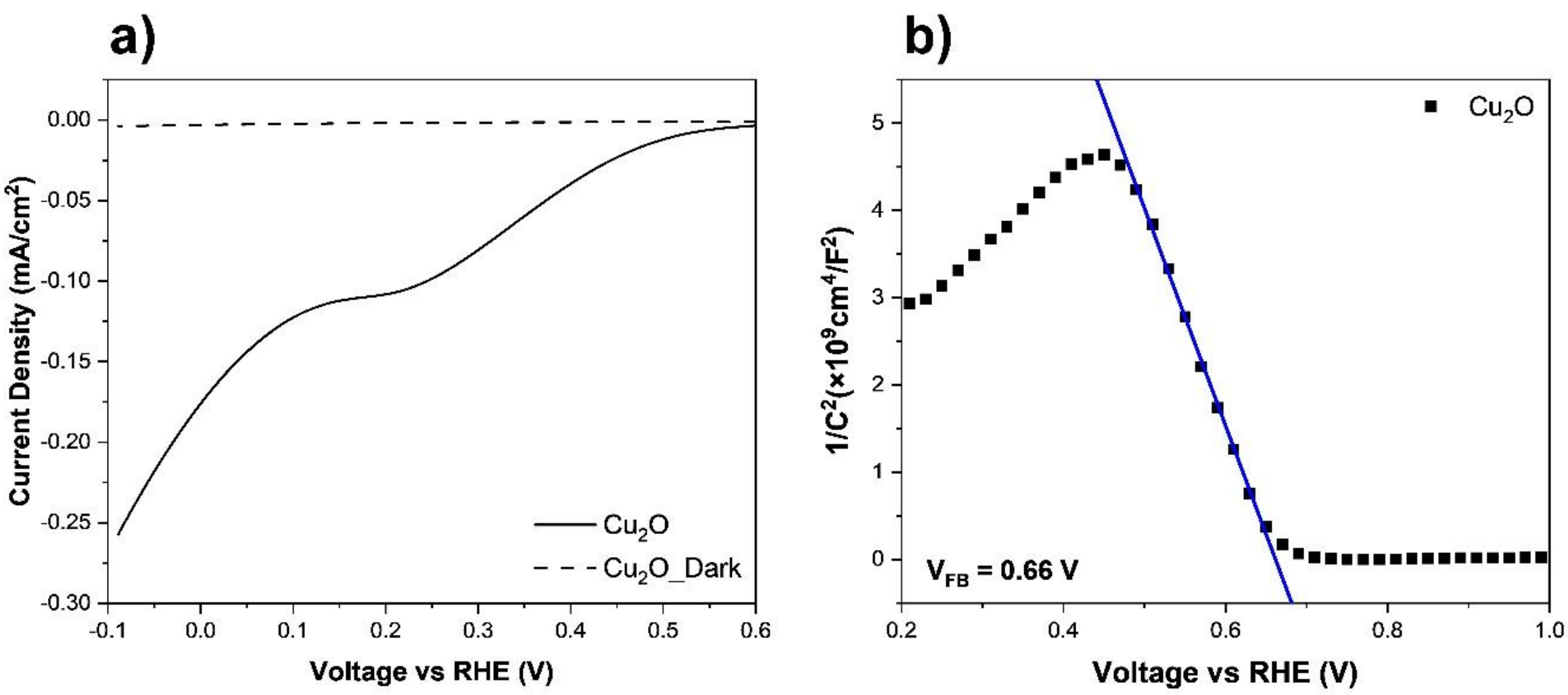

Figure S4. a) LSV curves of the Cu_2O photocathode under illumination (solid lines) and in the dark (dotted lines) in 0.1M Na_2SO_4 solution. b) The M-S plot of the Cu_2O photocathode was measured in 0.1M Na_2SO_4 solution at 100 Hz.

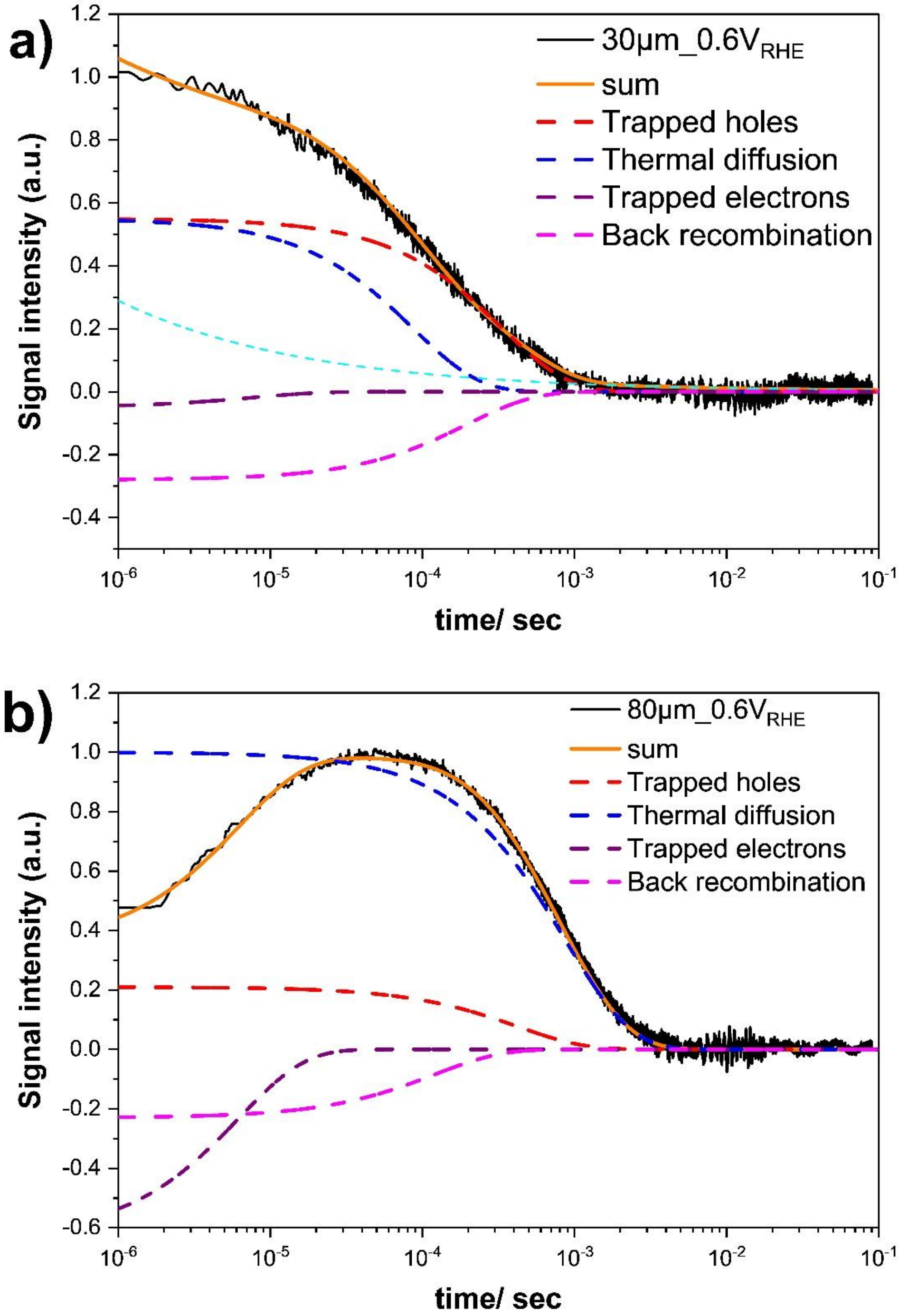

Figure S5. NF-HD-TG responses of Cu_2O photocathode at 0.6 V_{RHE} at the grating spacing of a) 30μm and b) 80μm with fitting curves.

Table S1. List of time constants of each component.

Λ (μm)	τ_1 [×10^{-4} s]	τ_2 [×10^{-4} s]	τ_3 [×10^{-6} s]	τ_4 [×10^{-6} s]
30	0.865	3.37	7.68	198
80	8.70	3.34	6.21	120

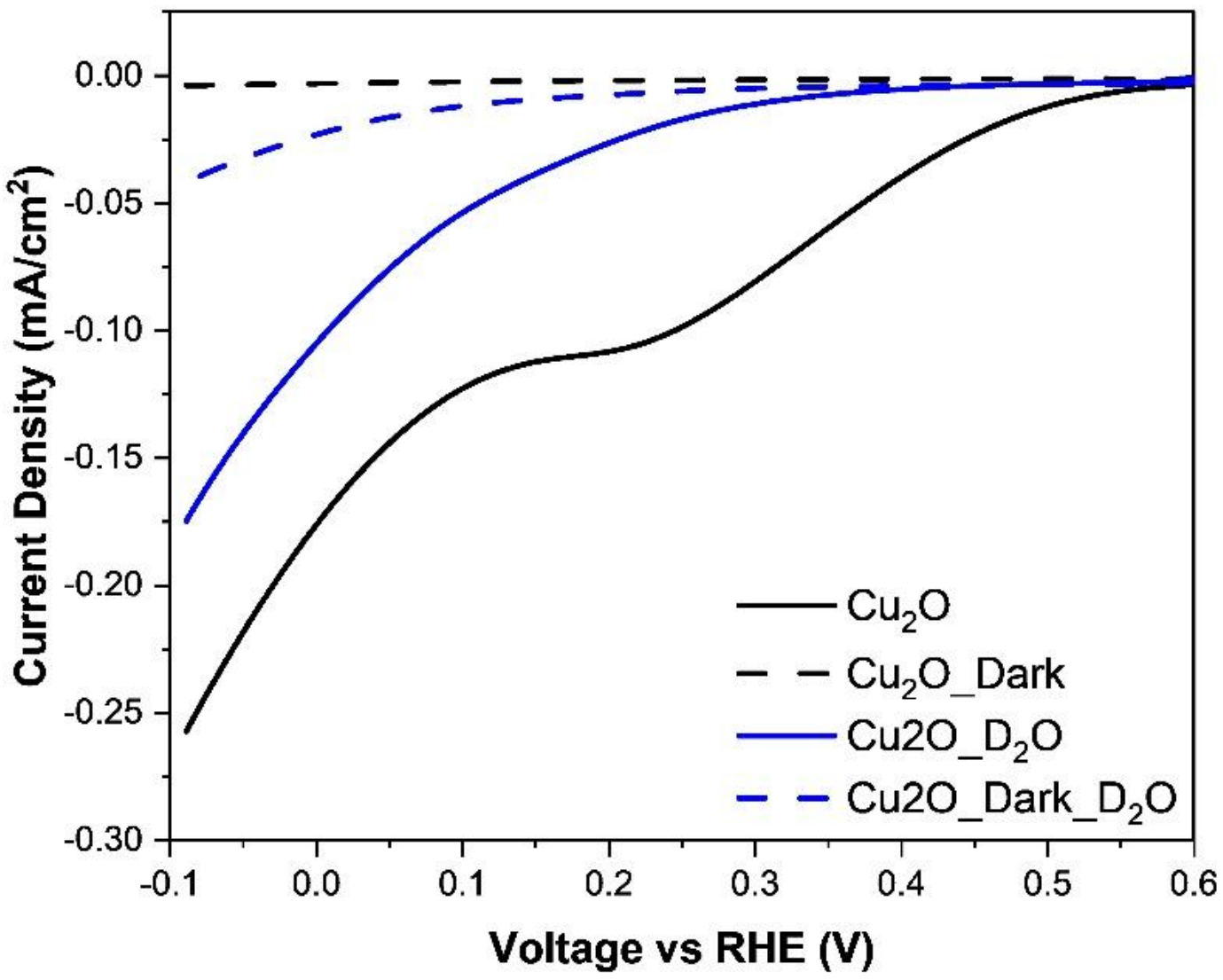

Figure S6. LSV curves of the Cu_2O photocathode under illumination (solid lines) and in the dark (dotted lines) measured in 0.1 M Na_2SO_4 solution, where the black and blue curves represent measurements in H_2O and D_2O electrolytes, respectively.

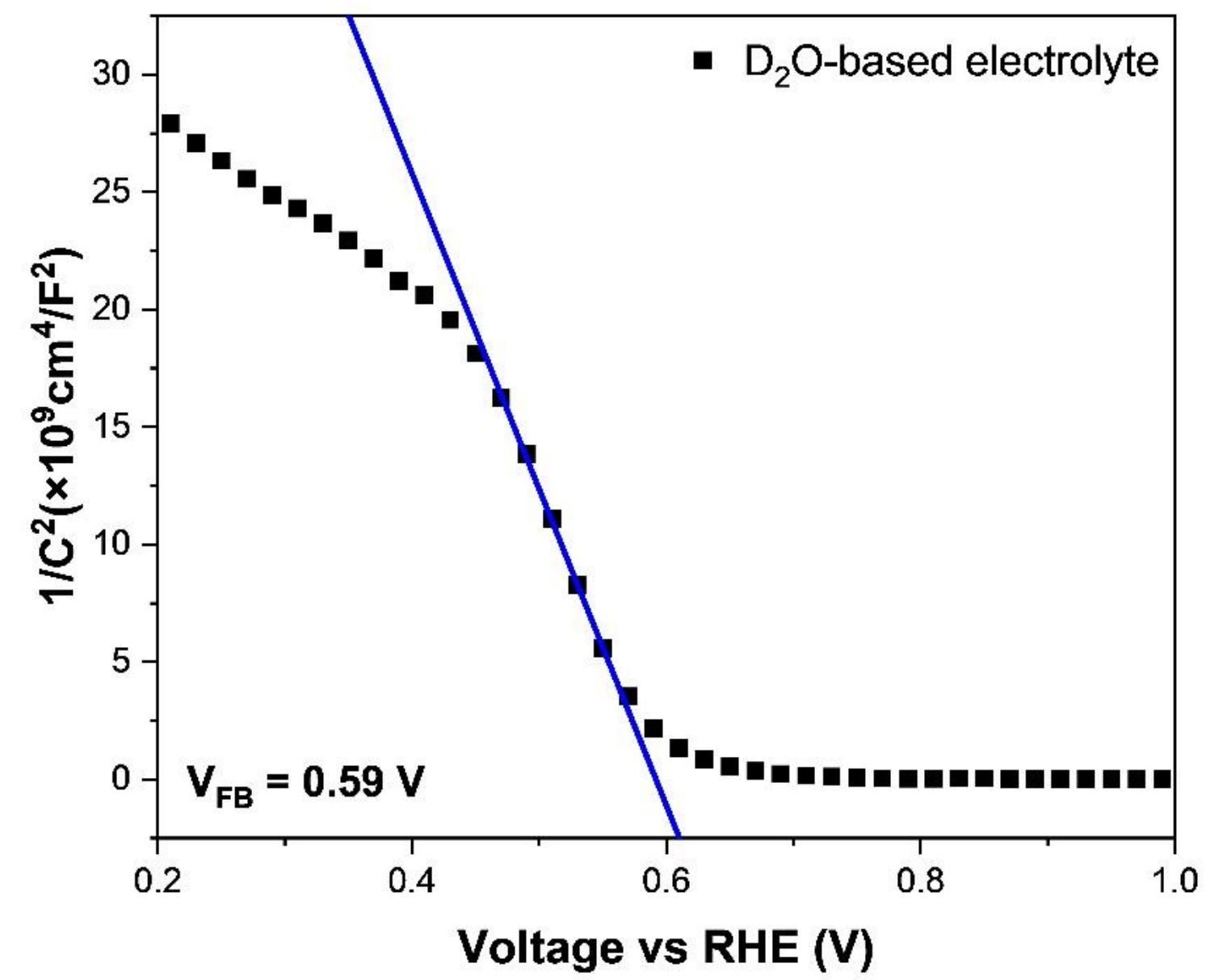

Figure S7. The M–S plot of the Cu_2O photocathode measured in a D_2O-based 0.1 M Na_2SO_4 electrolyte at 100 Hz.

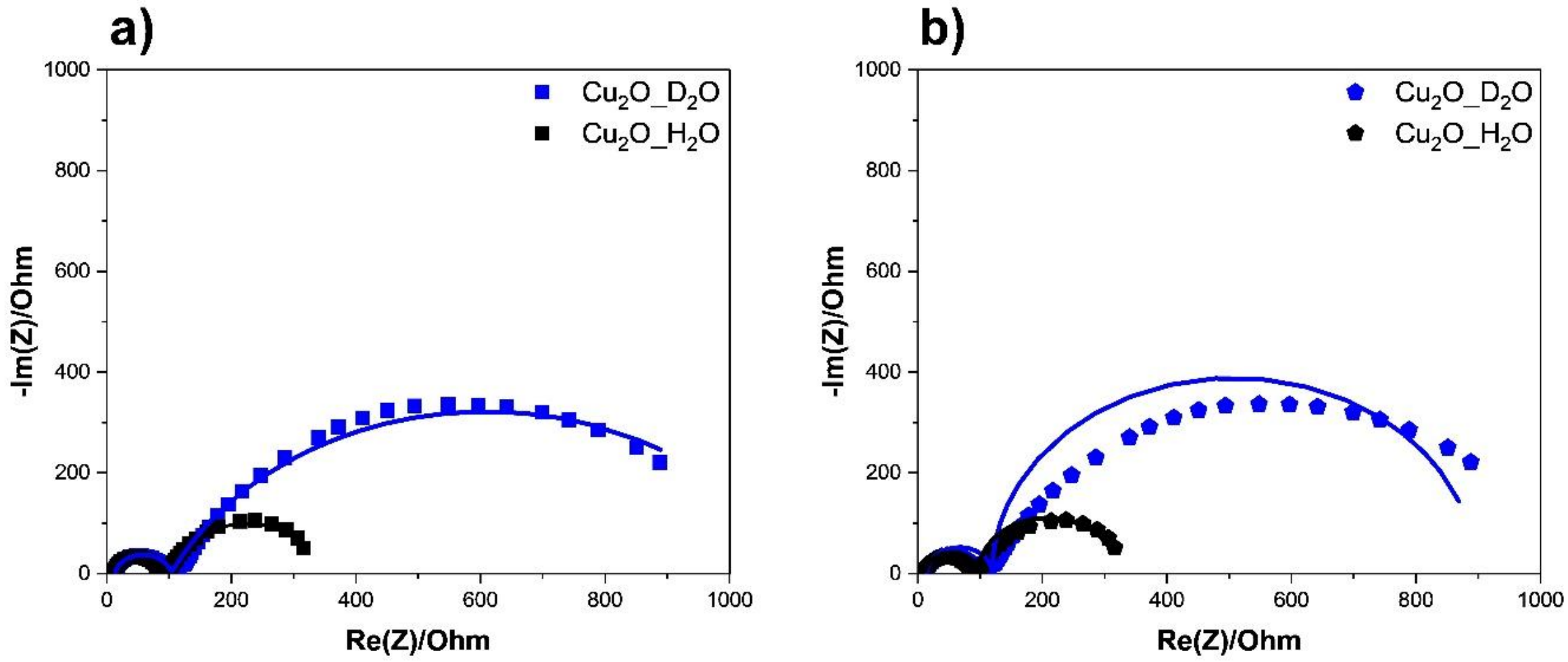

Figure S8. The circuit diagram and Nyquist plots of Cu_2O photocathode obtained through PEIS analysis at the $0V_{RHE}$ (Black: H_2O-based electrolyte, Blue: D_2O-based electrolyte), where a) the fitting was carried out using a constant phase element (CPE, Q) to account for non-ideal interfacial behavior, and b) the fitting was performed using an equivalent circuit with an ideal capacitance (C).

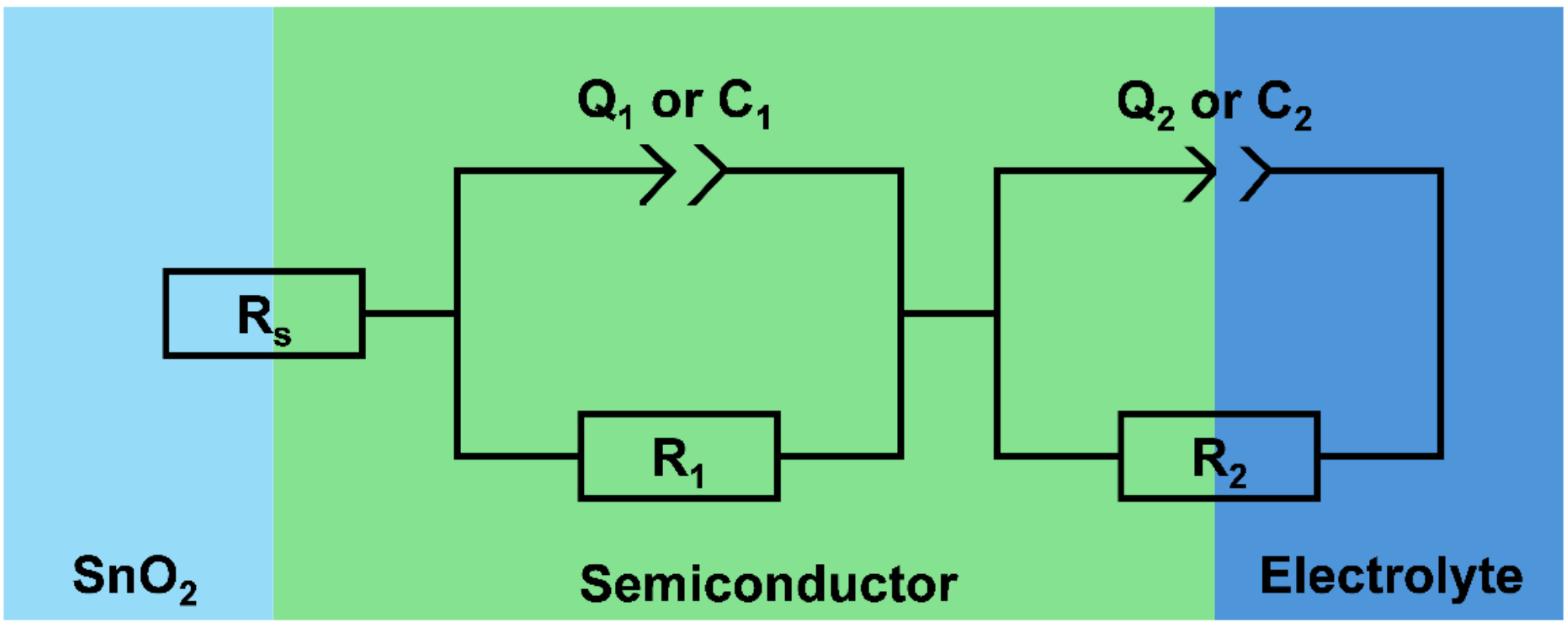

Figure S9. The circuit diagram of PEIS

Table S2. The PEIS fitting parameters of the Cu_2O photocathode at 0 V_{RHE} in H_2O- and D_2O-based electrolytes.

a) CPE (Q) model

	R_s [Ω]	R_1 [Ω]	Q_1 [×10^{-7}]	R_2 [Ω]	Q_2 [×10^{-3}]
H_2O-based electrolyte	11.1	83.6	1.42	264	1.28
D_2O-based electrolyte	12.2	93.9	3.04	1044	0.75

b) ideal capacitance (C) model

	R_s [Ω]	R_1 [Ω]	C_1 [×10^{-2} μF]	R_2 [Ω]	C_2 [×10^{2} μF]
H_2O-based electrolyte	12.1	85.1	4.51	223	8.95
D_2O-based electrolyte	14.3	105	4.55	777	4.77

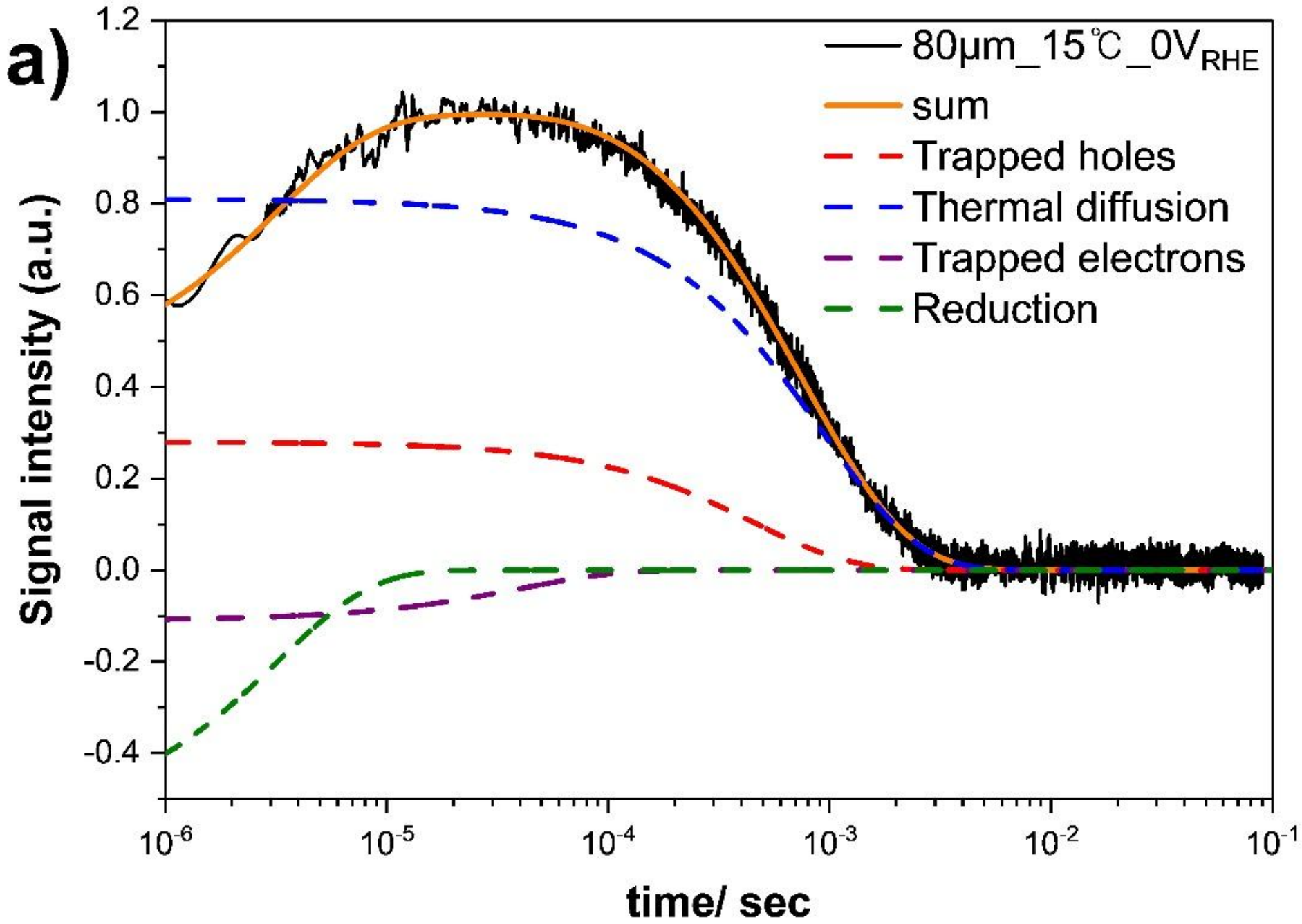
a)
80μm_15℃_0V$_{RHE}$
sum
Trapped holes
Thermal diffusion
Trapped electrons
Reduction
Signal intensity (a.u.)
time/ sec

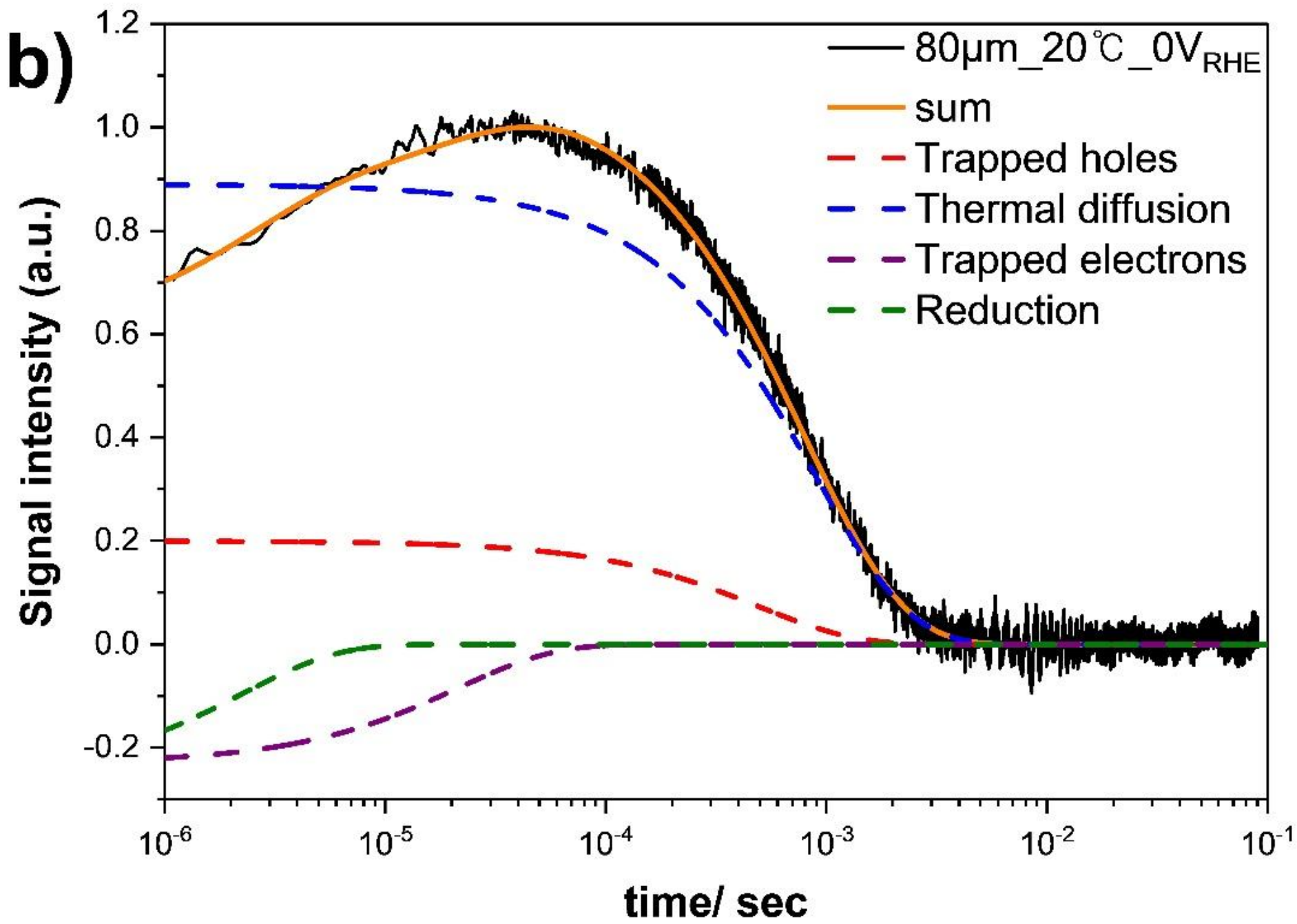
b)
80μm_20℃_0V$_{RHE}$
sum
Trapped holes
Thermal diffusion
Trapped electrons
Reduction
Signal intensity (a.u.)
time/ sec

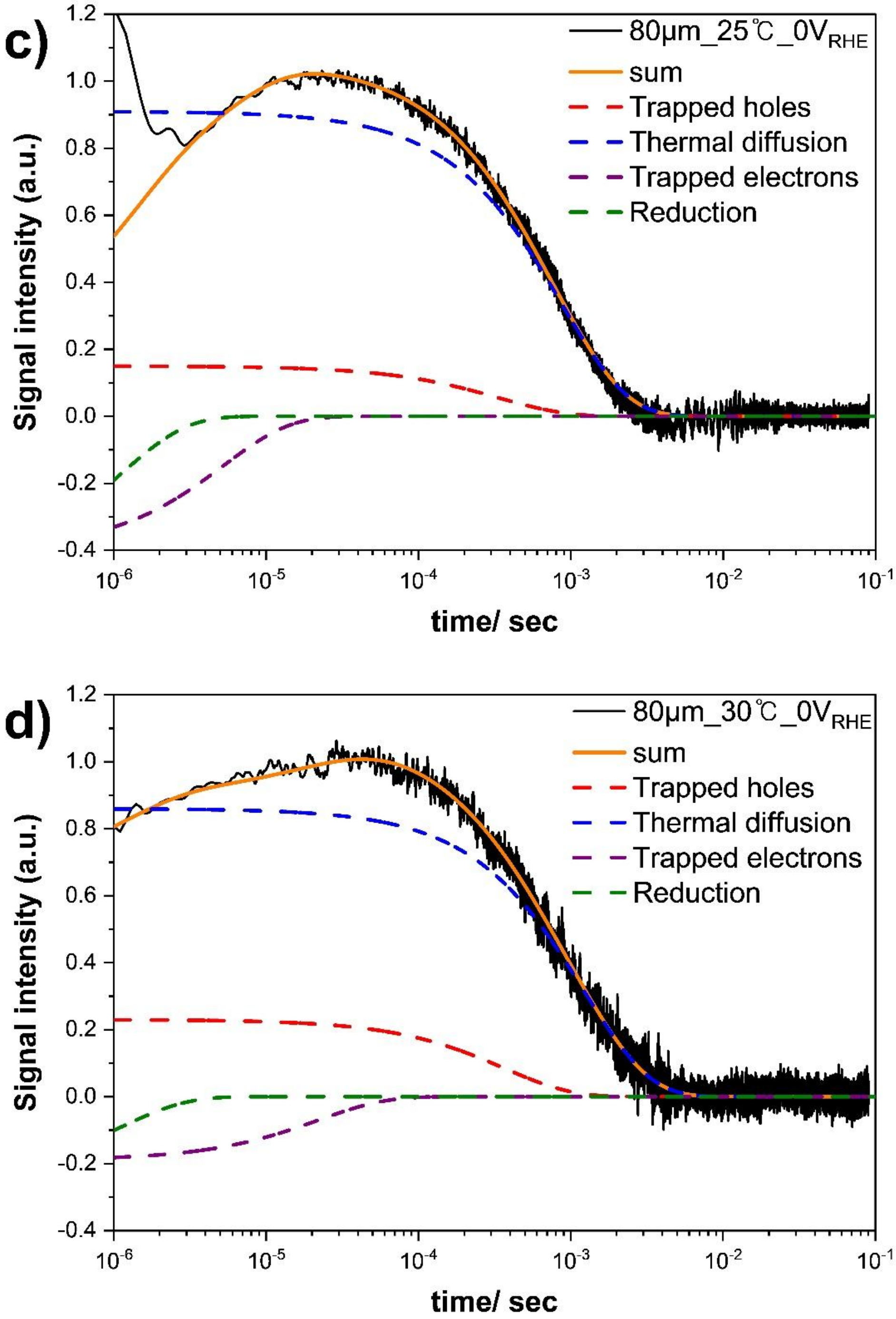
c)
80μm_25℃_0V_RHE
sum
Trapped holes
Thermal diffusion
Trapped electrons
Reduction
Signal intensity (a.u.)
time/ sec
d)
80μm_30℃_0V_RHE
sum
Trapped holes
Thermal diffusion
Trapped electrons
Reduction
Signal intensity (a.u.)
time/ sec

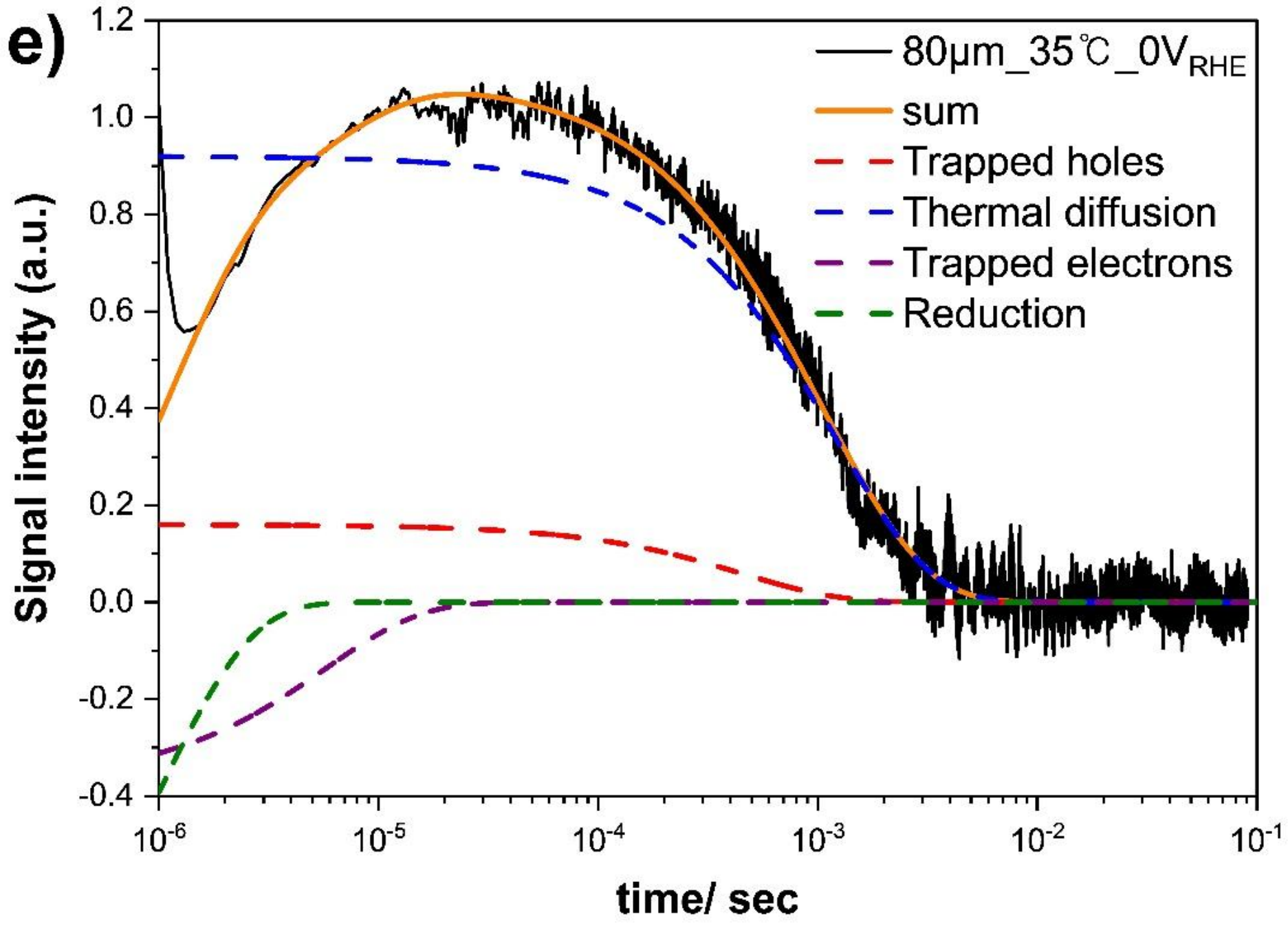

Figure S10. NF-HD-TG responses of the Cu_2O photocathode measured during first experiment at 0 V_{RHE} with the electrolyte under a) 15 °C, b) 20 °C, c) 25 °C, d) 30°C, and e) 35°C.

Table S3. List of time constants of each component in the first experiment.

	τ_1 [$\times 10^{-4}$ s]	τ_2 [$\times 10^{-4}$ s]	τ_3 [$\times 10^{-5}$ s]	τ_4 [$\times 10^{-6}$ s]
15 °C	9.40	4.58	4.01	3.18
20 °C	8.90	4.91	2.14	2.25
25 °C	8.74	3.35	0.524	1.11
30 °C	12.2	3.65	2.21	1.10
35 °C	12.1	4.54	0.578	0.97

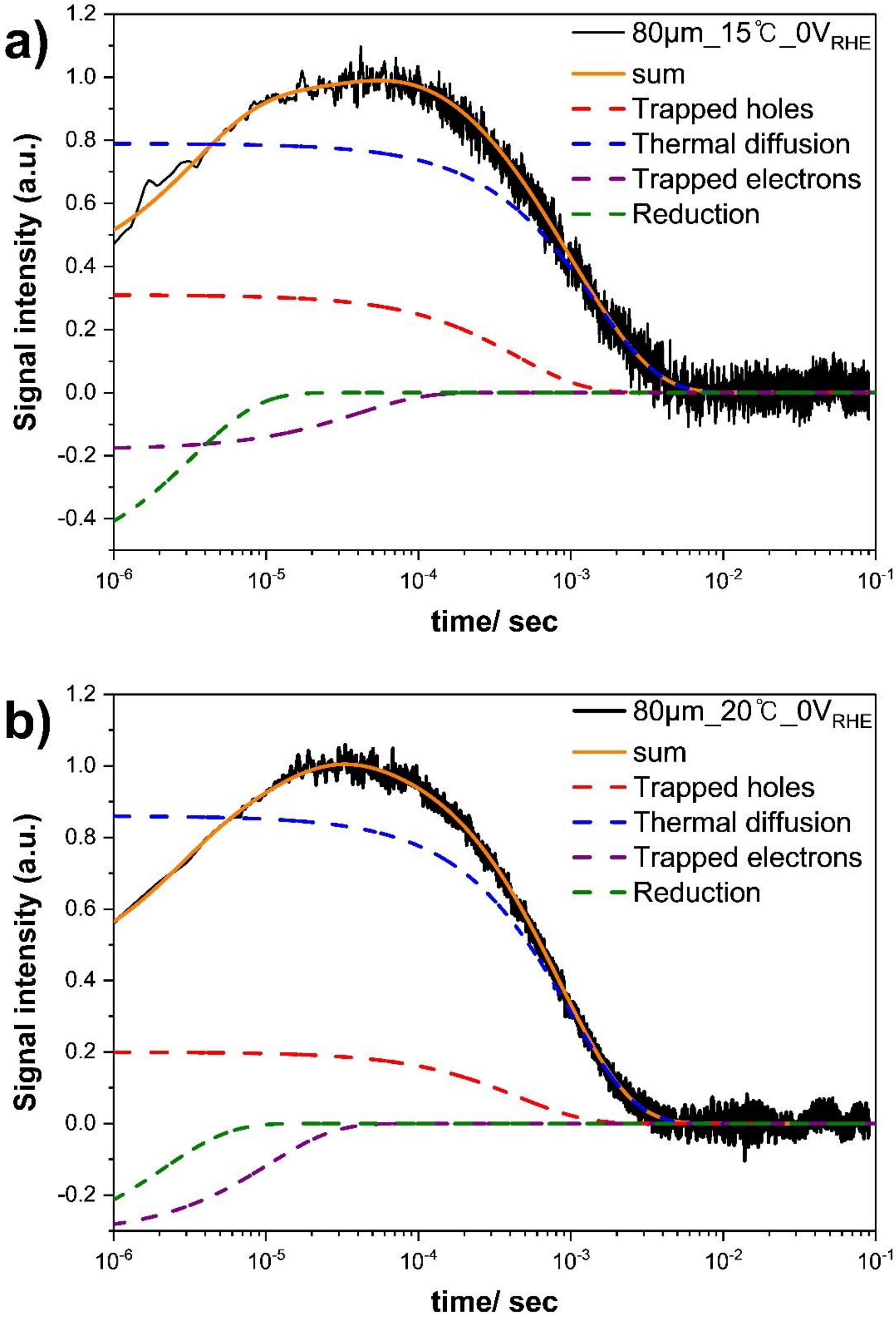
a)
80μm_15℃_0V_RHE
sum
Trapped holes
Thermal diffusion
Trapped electrons
Reduction
Signal intensity (a.u.)
time/ sec
b)
80μm_20℃_0V_RHE
sum
Trapped holes
Thermal diffusion
Trapped electrons
Reduction
Signal intensity (a.u.)
time/ sec

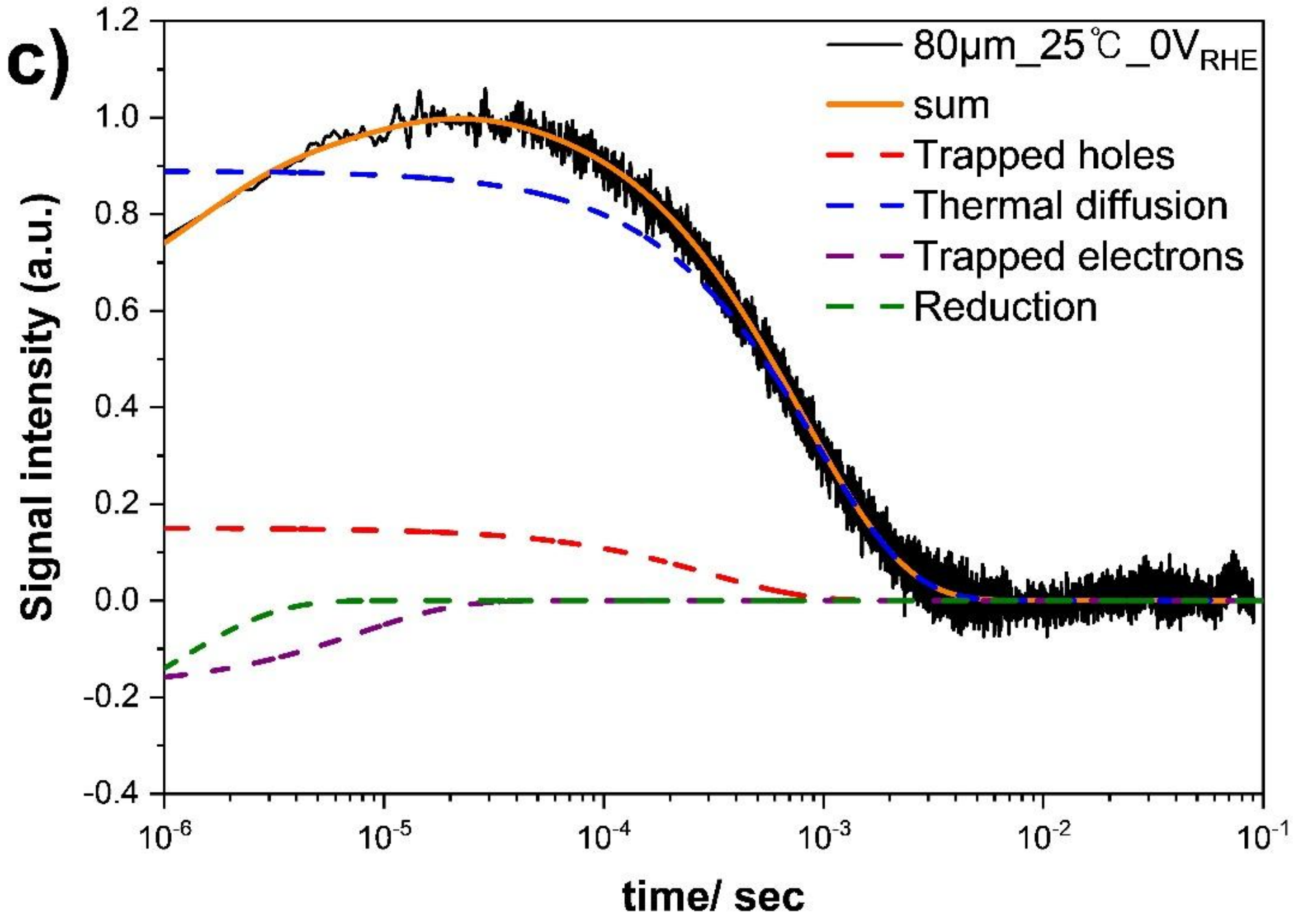
c)
80μm_25℃_0V_RHE
sum
Trapped holes
Thermal diffusion
Trapped electrons
Reduction
Signal intensity (a.u.)
time/ sec

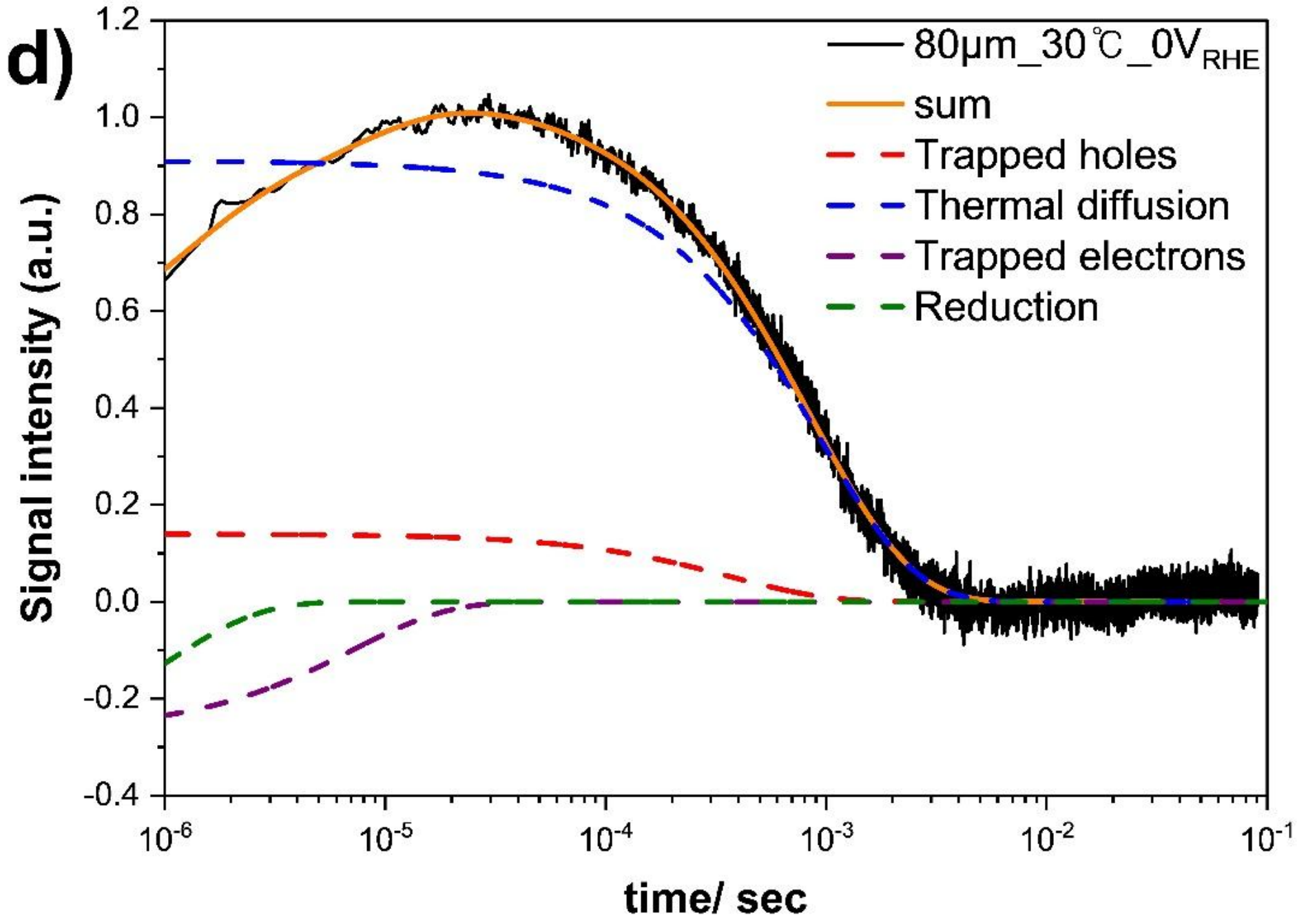
d)
80μm_30℃_0V_RHE
sum
Trapped holes
Thermal diffusion
Trapped electrons
Reduction
Signal intensity (a.u.)
time/ sec

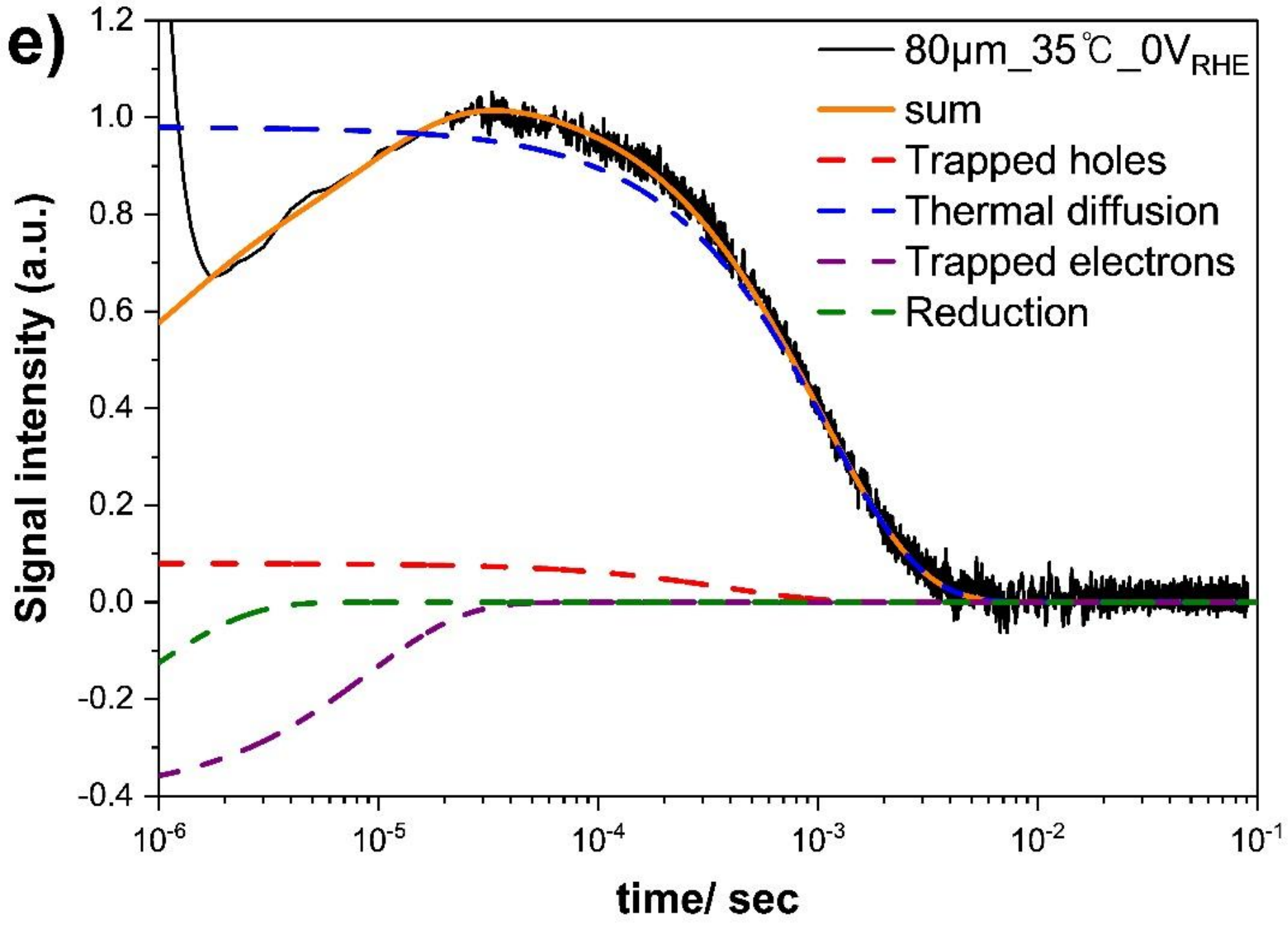

Figure S11. NF-HD-TG responses of the Cu_2O photocathode measured during second experiment at 0 V_{RHE} with the electrolyte under a) 15 °C, b) 20 °C, c) 25 °C, d) 30°C, and e) 35°C.

Table S4. List of time constants of each component in the second experiment.

	τ_1 [$\times10^{-4}$ s]	τ_2 [$\times10^{-4}$ s]	τ_3 [$\times10^{-5}$ s]	τ_4 [$\times10^{-6}$ s]
15 °C	14.4	4.48	3.98	3.33
20 °C	9.80	4.57	1.04	2.14
25 °C	9.24	3.04	0.78	1.21
30 °C	9.44	3.69	0.715	0.99
35 °C	11.04	3.89	0.901	0.97
